# Hydrogels as promising carriers for the delivery of food bioactive ingredients

**DOI:** 10.3389/fnut.2022.1006520

**Published:** 2022-09-27

**Authors:** Min Li, Xiaoqian He, Ran Zhao, Qixin Shi, Yingqun Nian, Bing Hu

**Affiliations:** ^1^College of Food Science and Technology, Nanjing Agricultural University, Nanjing, China; ^2^Guangdong Laboratory for Lingnan Modern Agriculture, South China Agricultural University, Guangzhou, China

**Keywords:** hydrogels, natural polymers, crosslinking, bioactive ingredients, adhesive, acidic stability, controlled release

## Abstract

The burden of public health challenges associated with the western dietary and living style is growing. Nutraceuticals have been paid increasing attentions due to their effects in promotion of health. However, in the gastrointestinal (GI) tract, the nutraceuticals suffer from not only the harsh acidic environment of the stomach and a variety of digestive enzymes, but also the antibacterial activity of intestinal bile salts and the action of protease from the gut microbiota. The amount of the nutraceuticals arriving at the sites in GI tract for absorption or exerting the bioactivities is always unfortunately limited, which puts forward high requirements for protection of nutraceuticals in a certain high contents during oral consumption. Hydrogels are three-dimensional polymeric porous networks formed by the cross-linking of polymer chains, which can hold huge amounts of water. Compared with other carries with the size in microscopic scale such as nanoparticle and microcapsules, hydrogels could be considered to be more suitable delivery systems in food due to their macroscopic bulk properties, adjustable viscoelasticity and large spatial structure for embedding nutraceuticals. Regarding to the applications in food, natural polymer-based hydrogels are commonly safe and popular due to their source with the appealing characteristics of affordability, biodegradability and biocompatibility. Although chemical crosslinking has been widely utilized in preparation of hydrogels, it prefers the physical crosslinking in the researches in food. The reasonable design for the structure of natural polymeric hydrogels is essential for seeking the favorable functionalities to apply in the delivery system, and it could be possible to obtain the enhanced adhesive property, acid stability, resistant to bile salt, and the controlled release behavior. The hydrogels prepared with proteins, polysaccharides or the mix of them to deliver the functional ingredients, mainly the phenolic components, vitamins, probiotics are discussed to obtain inspiration for the wide applications in delivery systems. Further efforts might be made in the *in situ* formation of hydrogels in GI tract through the interaction among food polymers and small-molecular ingredients, elevation of the loading contents of nutraceuticals in hydrogels, development of stomach adhesive hydrogels as well as targeting modification of gut microbiota by the hydrogels.

## Introduction

There is a continuing quest to safe and convenient approaches for overcoming the increasing burden in public health caused by the chronic diseases such as metabolic syndrome, obesity, diabetes and inflammation (1). Often this quest looks to dietary as a source of bioactive ingredients or inspirations, and often the journey leads to phytochemicals including phenolics, vitamins, polypeptides, polysaccharides and probiotics (2). Recently, these nutraceuticals have been demonstrated to have the bio-activities of anti-oxidant, anti-inflammation, anti-obesity, even anti-cancer (3, 4). In order to minimize the drug- and carrier-induced undesirable toxicity in the long-term oral consumption, no better-than-food-grade ingredients are currently suitable for the development of a desirable, sustainable and cost-effective strategy for the management of these chronic diseases.

However, the application of these nutraceuticals are still limited due to their low solubility, instability, low bio-accessibility and bioavailability, as well as the incompatibility with other components (5). In oral administration, the environment in gastrointestinal tract is a big challenge for the stability and consequent bioactivities of the nutraceuticals (6). The pH environment in GI tract is indeed complicated. The normal pH range for stomach acid is between 1.0 and 2.5. The pH value in small intestine is 6.0–7.0, while the mean pH in the distal ileum and in the body fluid at intercellular spaces between enterocytes is about 7.4. In addition, the presence of various digestive enzymes, such as pepsin and lipase in stomach, trypsin, amylase and lipase in small intestine, as well as the protease secreted by gut microbiota could degrade nutraceuticals. Furthermore, the nutraceuticals with living properties such as probiotics also suffer from the antibacterial activity of intestinal bile salts and the action of protease from the gut microbiota.

A wide range of delivery systems, such as nanoparticles, emulsions, microcapsules, gels have been developed for the encapsulation of the nutraceuticals to protect them and enhance their bioavailability and bioactivities (7). Among them, hydrogels are defined as the polymeric materials with three-dimensional porous networks formed by the cross-linking, physically and/or chemically, of polymer chains, which retain huge amounts of water in their spatial structure (8, 9). Compared with other carries with the size in microscopic scale from nanometer to micrometer, hydrogels could be considered to be more suitable delivery systems in food due to their macroscopic bulk properties, adjustable viscoelasticity and large spatial structure for embedding nutraceuticals. The GI tract environment is a double-edged sword for these oral delivery systems. On one hand, it is the challenge for the stability of these delivery systems. On the other hand, the variation of pH, the presence of digestive enzymes and the proteases of bacterials could be utilized as the stimulations for design of the carriers respond to environmental triggers.

Hydrogels prepared from nature sourced polymers, including proteins and polysaccharides, are essential for the applications in food industry due to the innate characteristics of natural polymers, such as readily available, low toxicity, biocompatibility and biodegradability. In addition to these attractive properties, it is believed that the natural polymer-based hydrogels with other desirable performance such as good swelling and preferred mechanical properties could be obtained when the proper design was carried out. Numerous studies have demonstrated that reasonable design of nature-sourced hydrogels plays a prominent role in modern food products, such as the prolonged shelf-life (10), the improved sensory textures (11), as well as the increased bioavailability of bioactive components (12). It is well–acknowledged that the bioactive components are easily available in food diets and there are growing interests in the hydrogels with the embedded bioactives due to the enhanced benefits to health. In the encapsulation systems constructed by hydrogels, the network structures containing lots of water or biological fluids provide the suitable environment for the bioactive components like polyphenols to disperse (13). Besides, the network of hydrogels can provide an isolated environment to protect the embedded components from harmful stimuli such as pH, heat and oxygen, and thus showing enhanced stability. On the other hand, the rational design of hydrogels enables them to respond to external conditions including pH, temperature and enzyme. Therefore, the oral delivery of hydrogels loading with bioactive ingredients may be an effective approach to realize the controlled release and the enhanced absorption in the GI tract, and thus achieving the improved bioavailability (14). The controlled release is always depend on diffusion of food bioactive ingredients, as well as swelling and erosion/breakup of the hydrogels.

In this review, the fundamentals about the classification of hydrogels, their major preparation methods based on physical and chemical crosslinking of natural polymers, as well as the important properties of hydrogels associated with their applications in delivery systems are introduced. Then the focus of present review was mainly placed on the targeted delivery of bioactive ingredients including phenolic components, vitamins, probiotics by different hydrogels in the simulated gastrointestinal tract. Further development of the *in situ* formation of hydrogels in GI tract, stomach targeted hydrogels, the interaction between hydrogels targeting intestine with gut microbiota are prospected as the future directions in the research field.

## Classification of hydrogels

**Figure 1:**
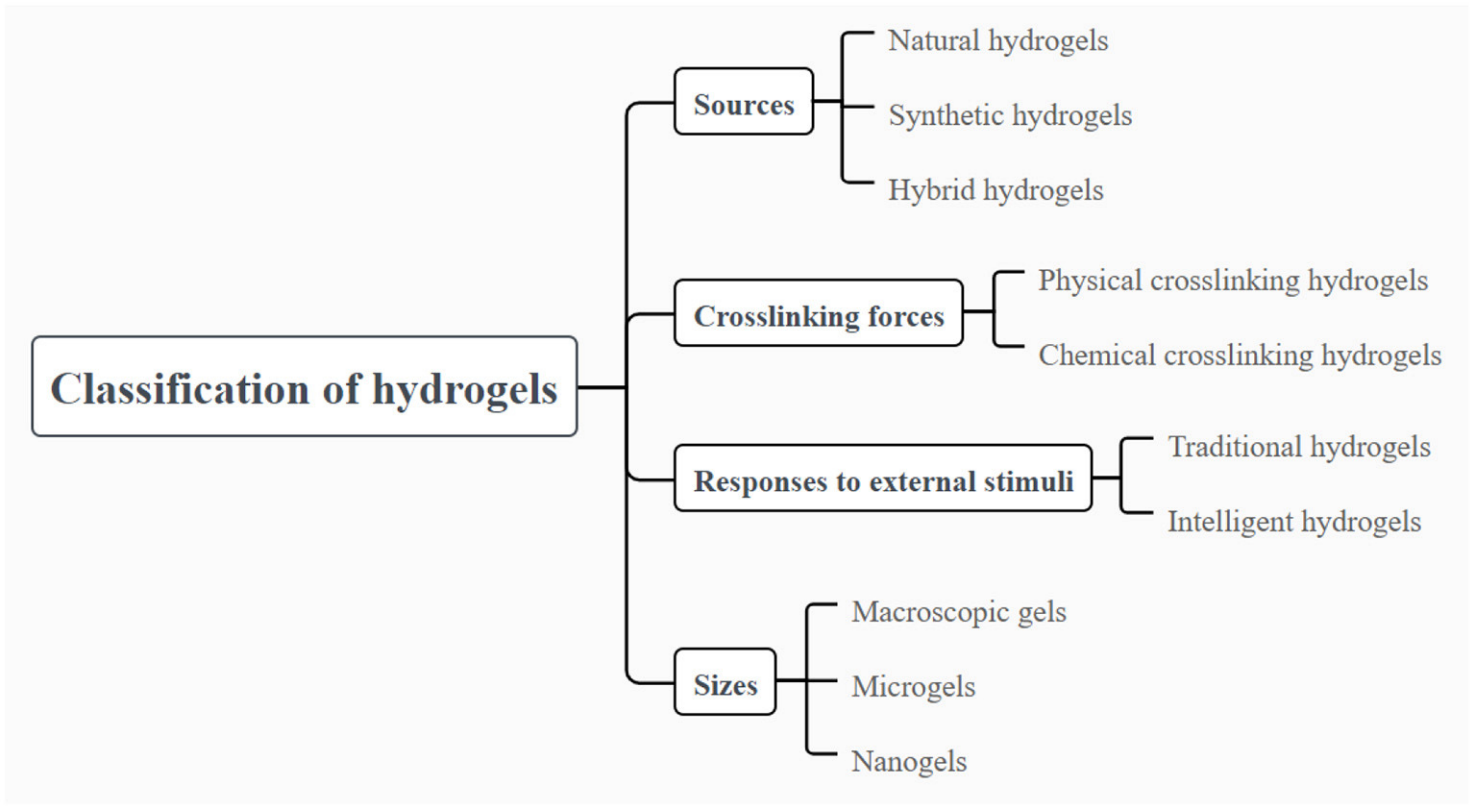
Classification of hydrogels based on different parameters of the polymer sources, differ crosslinking forces, responses to external stimuli and the size.

Hydrogels can be classified into different types based on different classification criteria. Here, we mainly focus on the source of polymers, type of crosslinking forces, response to external stimuli, size, and electrical charge to define the categories of hydrogels. The details of classification of hydrogels are summarized in Figure 1. The general characteristics, advantages and drawbacks of different categories of hydrogels are briefly summarized here for the reference. Detailed applications of the different type hydrogels in delivery of food nutraceuticals are presented in the following sections.

Hydrogels can be categorized as natural, synthetic and hybrid hydrogels with the combination of natural and synthetic polymers, displaying diverse molecular composition and different mechanical, physical and chemical properties. Natural polymers for preparing hydrogels are widely available in nature, which are mainly composed of polysaccharides and proteins such as alginic acid, chitosan, hyaluronic acid, cellulose, gelatin and soy proteins (15). Natural polymer-based hydrogels have attracted growing attentions because of their appealing biocompatibility, biodegradability and low toxicity. The most attractive property of natural polymer-based hydrogels is their biocompatibility. Natural polymer-based hydrogels appear to be extremely similar to extracellular matrix, and thus easily recognized by cells without causing adverse immune reactions (16). However, the mechanical properties of hydrogels synthesized from natural polymers are relatively weak and fragile, resulting in the restriction on the development of hydrogels to some extent in various areas. The hydrogels for encapsulation and delivery of nutraceuticals in food should be relatively weak and fragile, which to the best should be reversible. Thus, the hydrogels could be considered and taken as food, which could also be feasible for the digestion as well as release of the loading bioactive ingredients. On the other hand, synthetic polymers, widely used in the formation of hydrogels, mainly include polyvinyl alcohol, polyacrylamide, polyethylene glycol, Polyvinyl pyrrolidone (17). Unlike natural polymer-based hydrogels, synthetic polymer-based hydrogels exhibit outstanding physical and mechanical properties which are highly controlled, although they are usually lack of innate biological activity and biodegradability (18). Notably, it has been reported that synthetic polymers are commonly served as blending agents to strengthen the crosslinking between polymer chains, thus improving the mechanical strength and flexibility of the hydrogels (19). Consequently, in order to make full use of the advantages of natural and synthetic polymers, the blending of natural and synthetic polymers to fabricate hybrid hydrogel is an effective strategy to obtain hydrogels with the characteristics of safety, biocompatibility and controllable mechanical properties. In the applications in food, both of the natural and synthetic polymers should be further generally considered as safe.

In addition, hydrogels can be divided into two categories on account of the type of crosslinking forces between the polymer chains, including chemical crosslinking and physical crosslinking hydrogels. Chemical crosslinking used to construct irreversible hydrogels involves covalent bonding between polymer chains and exhibits highly stable and permanent chemical structure. In that case, the corresponding hydrogels are usually stable and not affected by the changes of external environment. On the contrary, physical crosslinking between polymer chains is induced by non-covalent forces such as hydrogen bonding, hydrophobic forces, Van der Waals interactions, chain entanglement and π-π stacking. Since these non-covalent forces are weak, the hydrogels prepared by physical crosslinking are susceptible to environmental stimuli. In general, physical crosslinking hydrogels can be prepared *via* processing techniques such as changing pH, temperature, pressure or adding ions (20). Because organic solvents or other reagents are not involved in the formation of physical crosslinking hydrogels, the hydrogels are suitable as carriers for delivery systems for food nutraceuticals.

Furthermore, various stimuli from external environment such as temperature, pH, light and ionic strength can prompt hydrogels to react with in varying degree. On the basis of the reaction intensity of hydrogels to external stimuli, the hydrogels can be classified into traditional and intelligent hydrogels. The traditional hydrogels cannot show any obvious changes to the external stimuli. In contrast, significant changes can be occurred in the internal network structures and functional properties when intelligent hydrogels are exposed to various stimuli like temperature, pH, chemical molecule, light, pressure, electric and magnetic fields (21, 22). In that case, hydrogels show the behaviors of swelling or shrinkage based on the introduction of hydrogen bonds, complexation, electrostatic interactions, as well as other non-covalent driving forces (23). Given that intelligent hydrogels exhibit the appealing swelling or shrinkage behaviors under the condition of external stimuli, it has attracted lots of interests especially in the aspect of enzyme catalysis, drug delivery and sensors (24–26). The GI tract environment is on one hand the challenge for the stability of hydrogels and on the other hand the stimulation that could be used for design of the environmental stimuli hydrogels.

Hydrogels can be designed into a variety of sizes and shapes to meet the demands of different applications. Based on the size, hydrogels can be classified into macroscopic gels, microgels and nanogels. Macroscopic gels possess the size varying from millimeters to centimeters, usually in the forms of columns, spheres, porous sponges, matrices, beads, films and fibers (27). In contrast, microgels and nanogels have smaller sizes, like micrometer scales or nanometer scales respectively. Generally, nanogels are paid more attention than macroscopic gels in parenteral administration because of their smaller size which makes them injectable into the body and provides a large surface area to achieve multivalent bioconjugation (28). For the oral administration, microgels and hydrogels are more suitable, but they are not appropriate to be applied in intravascular injection. Notably, nanogels can be utilized in intravascular injection to exert effective effects (16). Microfluidic technologies are advantage in the fabrication of the gels with the size in microscopic scale from nanometer to micrometer. The gel carriers with various size could be fabricated through changing the parameters, such as the diameter of the channel in the chip, the flowing speed of the micro-fluid, as well as the molecular weight, surface charge, concentration of the polymers. In food applications, hydrogels could be considered to be more suitable delivery systems than the carries with the size in microscopic scale from nanometer to micrometer, because of their macroscopic bulk properties and adjustable viscoelasticity. In addition, they are commonly considered as appealing candidates for the delivery of functional ingredients, due to the high water holding capacity and superior loading capacity (29, 30).

## Preparation of hydrogels

Various approaches are applied to prepare hydrogels with different sizes, shapes and composition to meet the applications in many aspects. Since the synthesis of hydrogel is fundamentally the result of covalent or non-covalent forces between polymer chains, the preparation methods of hydrogels can be divided into two categories, namely physical and chemical crosslinking methods. Usually, the appropriate experimental conditions is the key to prepare the hydrogels with desired properties. In addition, from the point view of food safety, since the hydrogels made from natural polymers have the excellent biological properties, the preparation methods are discussed mainly in terms of natural polymeric hydrogels.

### Chemical crosslinking

Chemical crosslinking refers to the formation of covalent bonding between polymer chains in the process of polymerization of low molecular weight monomers or cross-linking of polymers precursor (16). Covalent bonds, as bridges to connect the three dimensional network structure, are closely related to the type and number of the functional groups, as well as the reactivity of crosslinkers (31). In addition, they are known for the characteristics of strong stability and mechanical resistance. Thus, the structure and apparent morphology of the hydrogels prepared by chemical crosslinking exhibit excellent stability and even show the ability to maintain the state permanently. At the same time, the structures of hydrogels are diverse, mainly depending on the degree of crosslinking and the proportion of polymer and reagents (32). The major methods of chemical crosslinking for the synthesis of hydrogels include the use of cross-linkers, graft polymerization and radiation crosslinking.

#### The use of cross-linkers

At present, much attention is paid to the fabrication of chemical crosslinking hydrogels by applying cross-linkers to induce heterogeneous polymerization reactions. In the progress of preparation of hydrogels, the addition of external crosslinking molecules can lead to the generation of covalent bonds, which play an important role in strengthening water absorption and preventing the dissolution of hydrophilic polymer chains in an aqueous environment (33). There are two types of cross-linkers involved in the formation of covalent bonds, including natural and synthetic cross-linkers. Synthetic cross-linkers like aldehydes are extensively applied to conduct polymerization reactions with the presence of functional groups such as hydroxyl, carboxylic, and amine groups. Nevertheless, they are commonly toxic compounds, resulting in toxic side effects that are difficult to remove when participating in the process of the preparation of hydrogels.

From the perspective of safety, natural crosslinkers are preferred to synthetic crosslinkers for their safety and friendly to environment. Genipin, tannic acid, procyanidin, epigallocatechin gallate and citric acid are natural crosslinking agents commonly used for the preparation of biopolymer-based hydrogels (34). Over the past few years, genipin extracted from gardenia fruit has been widely employed as natural crosslinker to prompt the synthesis of hydrogels due to their desired biocompatibility and low-toxicity. Chitosan-based hydrogels prepared by using genipin as crosslinking agents showed an obvious increase in storage modulus, and the improved functional properties of hydrophilicity, swelling and biocompatibility (35). In a recent study, semi-interpenetrating hydrogels comprised of chitosan, genipin and polyethylene glycol were prepared, showing non-toxic effect on 3T3 cells with the addition of a certain concentration of genipin (36).

Apart from small biological molecules, enzymes such as transglutaminase and laccase can also participate in the crosslinking between biopolymer chains (37, 38). For instance, transglutaminase, which can catalyze acyl transfer reaction between γ-acylamino of glutamine residues and ε-amino of lysine, is capable of acting as a crosslinker to assist in the preparation of protein-based hydrogels combining with other processing techniques, which is an effective approach to obtain protein-based hydrogels with improved crosslinking density and functional properties (38).

#### Graft polymerization

Graft polymerization is a common method to form hydrogels triggered by chemical reagents or radiation (16). In this case, free radicals are generated by a monomer and thus grafted onto natural polymers to form hydrogels. It is noteworthy that grafting on a surface wrapped with a stronger support is an effective technique to obtain improved mechanical and biological features (8). Besides, this method has the advantages of mild reaction conditions, high grafting rate and easy accessibility. Grafting synthetic monomers over natural polysaccharides is a useful approach to strengthen the properties of polysaccharides-based hydrogels. It is reported that there are a large kind of polysaccharides used for the fabrication of various hydrogels by grafting polymerization, such as chitosan (39), cellulose (40), starch (41), and dextran (42). Especially, the modified starch-based hydrogels prepared by grafting polymerization attract a lot of interest because of their enhanced properties and expanded application areas. In addition, graft polymerization mostly involves cross-linked copolymers of acrylate and acrylic acid for the preparation of hydrogels of grafted starch-acrylic acid (8).

### Radiation crosslinking

Radiation crosslinking involves in high energy radiation to initiate polymer ionization and subsequent crosslinking to form network structures, such as gamma rays (43–45) and electron beams (45–47). It is an effective approach to fabricate novel hydrogels with the advantages of low cost, controlled operation, and no extra reagents. For example, in the absence of toxic initiators and chemical reagents, “soft” and highly elastic silk fibroin-based hydrogels prepared by chemical cross-linking reactions triggered by gamma-ray irradiation within and between molecular chains were investigated. A series of cell experiments showed that the silk fibroin-based hydrogels with different mechanical strength could stimulate the expression of specific genes in different differentiation directions of cells, suggesting its application prospect in tissue engineering (44).

The hydrogels prepared with suitable chemical-crosslinking methods that could ensure safety are useful for enhancing the strength and toughness. The strength and toughness of hydrogels are in particular important for their adjustable chewing sensation. The release of the embedded nutricueticals in the chemical-crosslinking hydrogels could be realized *via* the destruction of the structure by chewing in mouth, digestion in stomach and intestine, or the enzyme hydrolysis by the gut microbiota.

### Physical crosslinking

In recent decades, physical crosslinking hydrogels have become the focus of many researchers due to their richer properties and applications than chemical crosslinking hydrogels. Most importantly, the hydrogels formed by physical crosslinking are usually non-toxic because no chemical reagents are involved in the preparation of hydrogels (48). Therefore, it is possible to produce safe and eco-friendly natural polymer-based hydrogels, and they have attracted huge attention especially in the food industry.

Physical crosslinking involves the connection of non-covalent forces to form reversible hydrogels. The intensity of non-covalent forces between polymer chains has a great influence on the structure of hydrogel. When the number of non-covalent forces are enough, a stiff hydrogel can be obtained, whereas a weak hydrogel can be prepared with the limited non-covalent forces (31). To obtain the desired characteristics of physical crosslinking hydrogels, three crucial factors should be taken into consideration: pH, temperature and the concentration of polymer (49). In this regard, various methods of physical crosslinking have been reported to prepare natural polymer-based hydrogels, mainly including heating/cooling, freeze-thaw cycles and complex coacervation.

#### Heating/cooling

Polysaccharides or proteins can form hydrogels by heating or cooling polymer solutions. Some polysaccharides can form hydrogels by simply heating or cooling polymer solutions, which is closely related to the structure of polysaccharides (50). In general, protein-based gels attract more attention because of their complex hierarchical structures. Specially, collagen/gelatin has the distinctive structure of three helices, which can form gels by cooling the hot solution. Nevertheless, for globular proteins, heating is the main route to form protein-based hydrogels. Except for casein and gelatin, most of food proteins are globular proteins such as whey proteins, soy proteins and egg white proteins, which can be heated at appropriate pH or/and salt concentration to induce the formation of hydrogels (51, 52). Note that globular proteins are easily to be denatured and unfolded to form the protein aggregates during thermal treatment for a period of time at acidic condition, and subsequent cold-set gels can be formed by changing pH or salt concentrations at room temperature (53). The protein aggregates mainly include particulate, strand and amyloid fibrils as building blocks of protein-based hydrogels, and the category of them mainly depend on the conditions in the formation of protein-based gels (54). Particulate often consist of large aggregates with the size of wide range from several hundreds of nanometers to more than 1 μm (55). Strand aggregates possess the diameter equivalent to the diameter of one or more protein molecules (55). Amyloid fibrils, known for unique cross-β secondary structure, have a diameter of several nanometers and the length of a few micrometers, and possess lots of attractive properties such as extreme aspect ratios, high hardness and low allergenicity (56). Among them, strands and amyloid fibrils are commonly used to construct cold-set gels, which possess better properties than typical heat-set gels including higher mechanical strength, improved water absorption and lower concentration for gelation (57). For example, β-lactoglobulin and whey protein isolate could be induced to form amyloid fibrils by heating, then cold-set gels were synthesized by the effects of pH or addition of salt which involve the connect of electrostatic interactions (58, 59). It is well–worth noting that the cold-set gels are important materials for the encapsulation of bioactive compounds which are extremely sensitive to temperature.

#### Freeze-thaw cycles

**Figure 2:**
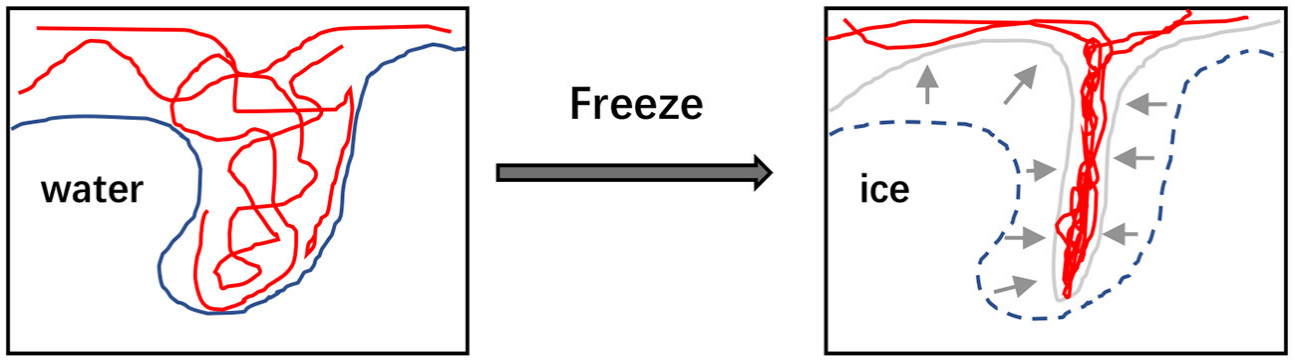
The illustration that freezethaw cycles would reform the microstructure of polymer hydrogels.

It is a useful strategy to establish the hydrogels with the improved performance through the process of freeze-thaw cycles. This method is based on the principle of phase separation to form microcrystalline zones inside the materials, and then three-dimensional network structures formed by using microcrystalline zone as cross-linking center to prepare hydrogels (Figure 2) (60). Most of polysaccharides can be used to prepare hydrogels by freeze-thaw cycles without the presence of extra reagents (60). The formation of crosslinked structures between polymer chains mainly depends on the frozen state, and thus the composition of polymers, the number of freezing cycles and the freezing temperature are the major factors influencing the properties of hydrogel products. Generally, the prepared hydrogels show more stable structure and perform stronger elasticity with the increased number of freeze-thaw cycles. Furthermore, higher porosity and smaller pores of hydrogels can be obtained *via* increasing the freezing time or reducing the freezing temperature (61). For the gelation process of locust bean gum, more than three times of freeze-thaw cycles led to obvious increase in the dynamic rigidity of the hydrogel, and the higher storage modulus of hydrogel could be tested with the faster freezing speed (62).

Furthermore, apart from the relative conditions with the freeze-thaw cycles, other conditions such as pH, soluble additives and the composition of biopolymers are important factors to affect the gelling properties. Alginate dissolved in pH 4.0 and 3.5 solutions could form gels after the freeze-thaw treatment. Compared with the gel without the treatment, the one formed after the freeze-thaw cycles showed 100 times increase in the storage modulus (63). Besides, it was found that the gel strength decreased with the increase of NaCl concentration, which might be related to the decreased degree of crosslinking (63). Additionally, chitosan-polyvinyl alcohol hydrogels prepared by freeze-thaw cycles were investigated, which showed controlled swelling and mechanical properties with the change of freezing conditions (61). Meanwhile, it was found that drug release time of chitosan-polyvinyl alcohol hydrogel loaded with drug was up to 30 h (61). Recently, the characteristics of high water absorption, swelling, mechanical and rheological properties were also observed in chitin-glucan complex hydrogels fabricated by freezing and thawing (64).

#### Complex coacervation

Complex coacervation involves the mix of polyelectrolytes with opposite charges, and then ionic interactions act as the main non-covalent forces to drive the self-assembly of polymers to form hydrogels. In general, the ionic interactions can commonly take place between proteins (65) or polysaccharides (66), even between protein and polysaccharide (67, 68). For the polyelectrolyte complex hydrogel, aside from changing pH or ionic concentration, it is an effective way to obtain the tunable characteristics of hydrogel by changing the proportion of polyelectrolytes. Recently, there was a study that revealed a novel polyelectrolyte complex hydrogel readily synthesized by self-assembly of salecan and N, N, N-trimethyl chitosan, and discovered that the behavior of self-assembly was induced by electrostatic interaction between polysaccharides (68). Salecan belongs to the family of β-dextran. Meanwhile, it was indicated that the swelling, morphology and rheology properties of the complex hydrogel could be varied by changing the proportion of the two composite polysaccharides (69). In addition, with the condition of pH near the isoelectric point of the protein, the mix of protein and polysaccharide can also lead to the formation of hydrogel which is primarily driven by electrostatic interaction, showing many favorable properties such as strong water-holding capacity (70, 71).

## The functionalities of hydrogels

It is important to endow hydrogels with the favourable functional properties, which is highly related to their applications in wide range field. It is well-known that water absorption, swelling, mechanical strength, biocompatibility and biodegradability are important properties of hydrogels which are highly correlated with the application of hydrogels in delivery systems. In general, these properties are mainly affected by several parameters including the chemical structure of polymers, the degree of crosslinking between polymeric chains and the external stimuli (72).

One of the important properties of hydrogels is the capability of high water absorption and thus also fulfilling satiety requirements for low caloric intake in food product design. It is considered to have the close association with the swelling capacity. This performance can be explained with the presence of available hydrophilic moieties in polymeric networks, such as hydroxyl, amine and sulfate groups. The structures of the crosslinked network can effectively control the ability of water absorption as well as assisting in the maintenance of swelling equilibrium (73).

The swelling behavior is an important property of hydrogel, which could determine the release profile of the embedded nutraceuticals from hydrogels. Hydrogel swelling suffers the restriction of elastic forces of the network and is closely related to the compatibility between polymer chains and water molecules (74). On one hand, the crosslinking and charge densities of polymers are the key factors determining the elastic behavior of hydrogels, and thus affecting the equilibrium swelling degree. The higher density of crosslinking often causes the lower swelling ratio, whereas the higher swelling ratio can be obtained with the increased hydrophilic groups. On the other hand, the specific stimuli such as temperature and pH can cause the shrinking or swelling of network structures to change the swelling degree. An increase in temperature may be benefit to the improvement of swelling capacity (75). Under the influence of the external stimuli, the change of swelling behavior of hydrogels may be conducive to the controlled release of the incorporated nutraceuticals in the delivery system.

The mechanical properties of hydrogels are highly associated with chewing sensation, the digestion and destruction rate in GI tract, and consequently the fermentation by gut microbiota. They are usually assessed by the elastic modulus and viscous modulus, so that the hydrogels are also called viscoelastic materials. The desired mechanical properties of hydrogels can be obtained by incorporating specific polymers, the change of crosslinking degree and the use of crosslinkers or nanocomposites (76–78). Generally, with the increase of the degree of crosslinking, a hydrogel with strong mechanical strength can be generated (79). Nevertheless, the excessive crosslinking may be harmful to maintain the stability of network structures, leading to low elasticity and brittleness. Therefore, in the process of the preparation of hydrogels, the optimal density of crosslinking is expected to occur to obtain the moderate mechanical strength and elasticity. It is worth mentioning that highly stretchable and tough hydrogels with improved tensile strength and elasticity have attracted much attention because of their potential applications in biomedical field such as wound healing, tissue culture and drug delivery (80–82). Besides, when incorporated with electronic components, the stretchable hydrogels can be used to create biomedical devices like wearable electronics (83, 84).

Moreover, the increasing focus is put on the polymers with the characteristic of biocompatibility and biodegradability. Biocompatibility of hydrogels is an extremely important characteristic showing the potential to be accepted by the body and not causing significant inflammatory. The nature-sourced polymers exhibit excellent biocompatibility (85). Thus, the hydrogels composed of food polymers are harmless when they are applied into the body system. Biodegradability means the polymers could be metabolized into the degradation products generally recognized as safe (86). To the best of our knowledge, natural polymers are popular as the alternative materials to suit for the synthesis of products with biodegradability, and thus meet the demands of sustainable development and friendly to environment. Especially, hydrogels composed of proteins, polysaccharides or the mix of them are widely studied to seek biodegradable materials. Besides, biodegradable hydrogel plays an important role in the delivery system, as the controlled degradation can effectively help achieve the controlled release especially for the delivery of large molecules (17). The controlled release of embedded ingredients in hydrogel can be in progress by controlling the rate of degradation.

## Application of hydrogels in the delivery of bioactive ingredients

**Figure 3:**
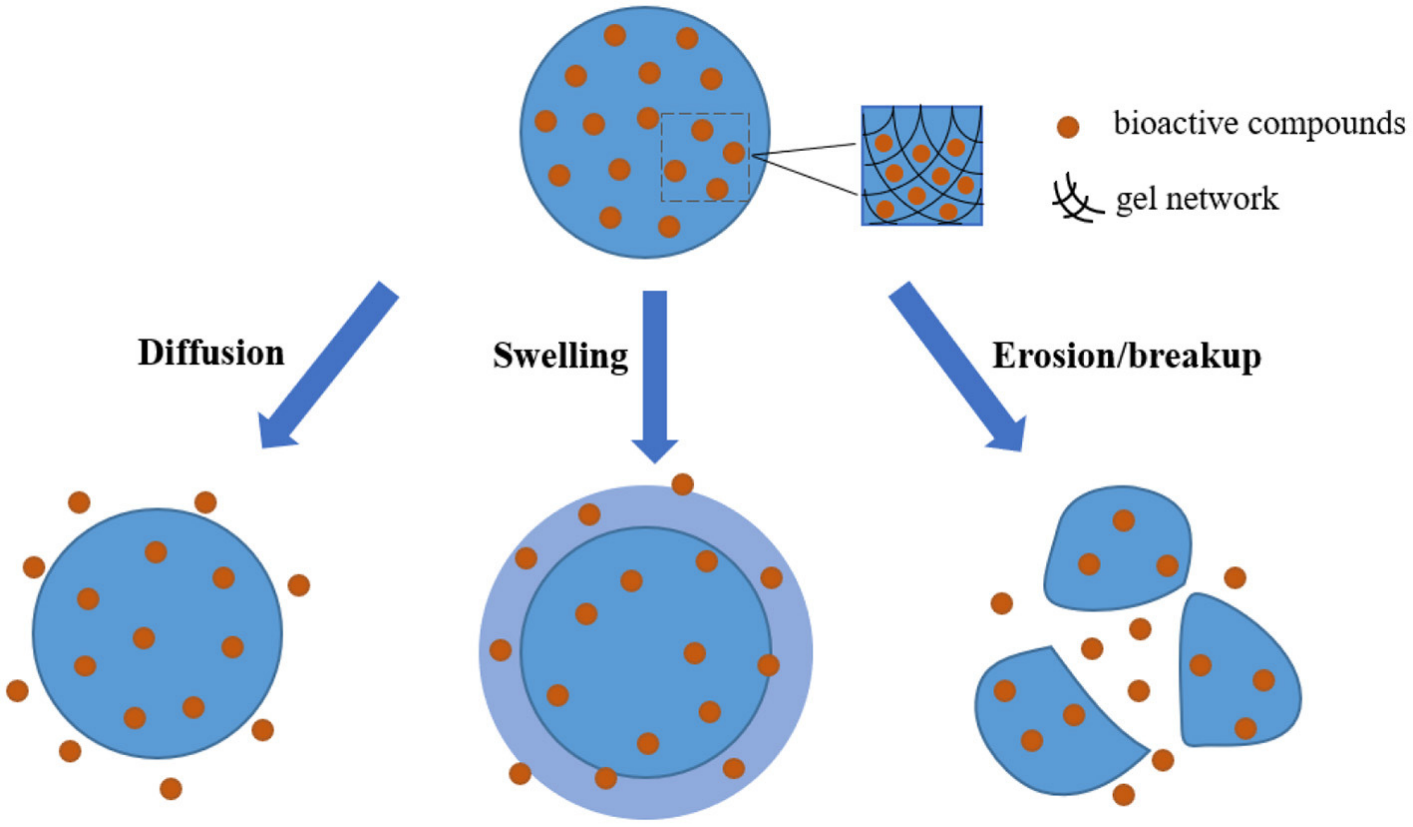
Schematic illustration of the release behavior of bioactive compounds when hydrogels as carriers are applied in the delivery systems.

Hydrogels have the unique three-dimensional networks containing a lot of water, which can provide a suitable environment for the loading of some substances, and thus they have great potential in serving as carriers for the delivery of effective substances. On the other hand, as increasing demand for health, there is no doubt that healthy diets play an important role in preventing or alleviating diseases, and this effect mainly depends on the bioactive ingredients in diets. Nevertheless, when these bioactive substances in diets simply enter the human body by the way of food intake, it is actually difficult to exert their functional activities because of their instability and low bioavailability. It is worth noting that the networks of hydrogel can not only protect the embedded components from external stimuli but also provide the possibility to achieve the controlled release at specific site within the gastrointestinal tract, and thus showing the enhanced stability and bioavailability. In general, the release behavior of bioactive ingredients uniformly dispersed in the hydrogel matrix is mainly based on the mechanisms by diffusion, swelling, as well as erosion/breakup (87–89), and the schematic illustration is showed in Figure 3.

**Figure 4:**
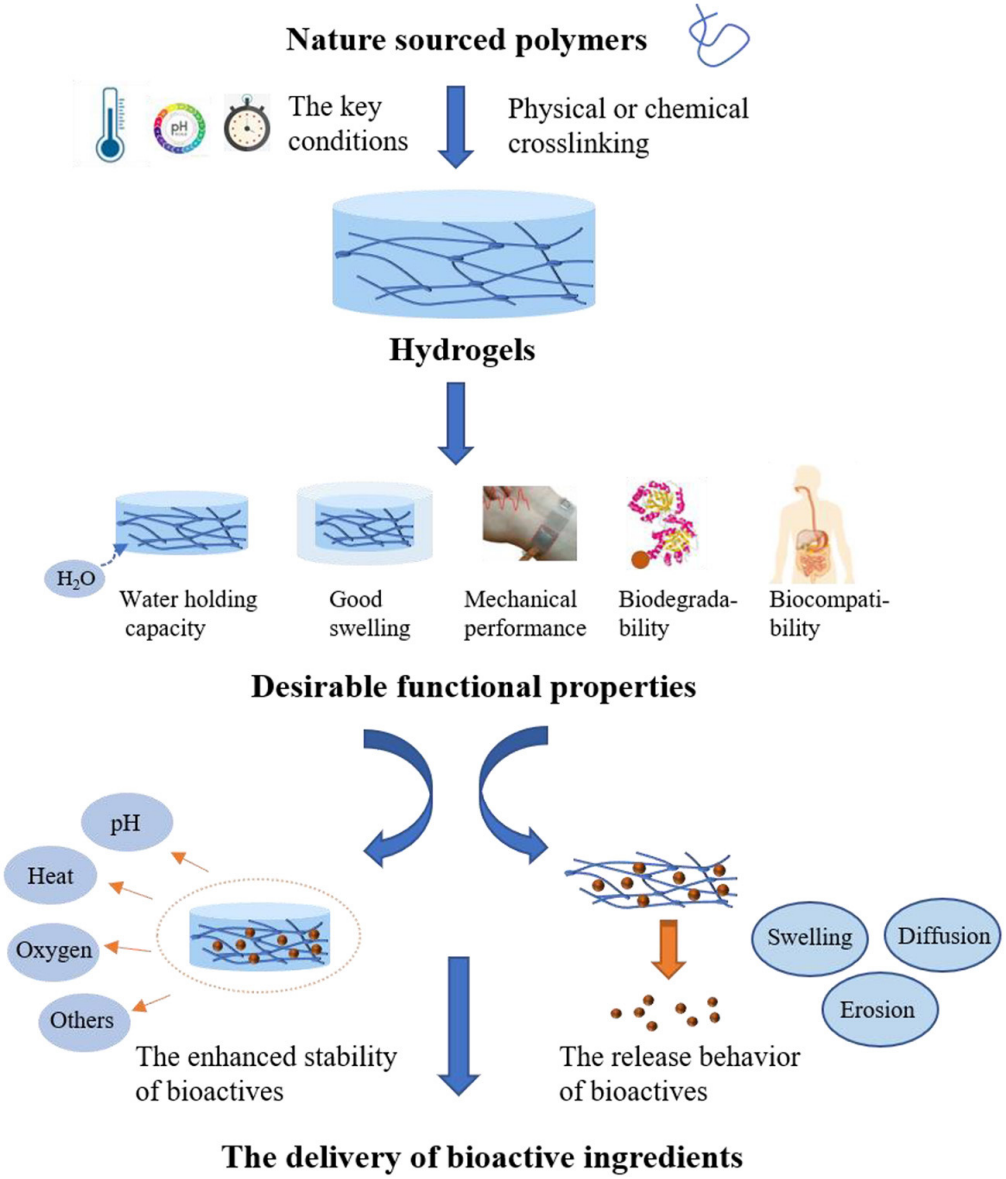
The factors associated with hydrogels as carriers of bioactive ingredients to realize the effective delivery.

It is well–known that bioactive ingredients embedded in hydrogels include hydrophilic or hydrophobic bioactive substances, mainly involving vitamins, polyphenols, carotenoids, probiotics, fatty acids and minerals, which have been broadly studied for the encapsulation with various delivery systems (12, 90). Meanwhile, the forms of hydrogels used to encapsulate bioactive molecules are diverse, mainly involving microparticles, nanoparticles, beads and emulsions-filled (91). Based on the contents described above in this review, the factors associated with the capabilities of hydrogels acting as carriers of bioactive ingredients to realize the effective delivery could be summarized in Figure 4.

Proteins, as the nutritional composition in diet, are composed of numerous amino acids with lots of functional groups. They can participate in the encapsulation of bioactive molecules because their functional groups are capable of interacting with molecules to promote the formation of hydrogels. In general, heat treatment, pressure, acid, salts, or enzymatic catalysis are main approaches to lead to the process of gelation. Proteins such as gelatin, whey protein and egg white protein are widely used to form hydrogels to encapsulate varieties of molecules to realize nutrition delivery (89).

Polysaccharides abundant in nature are non-toxic, biocompatible, and biodegradable biopolymers, containing a large number of hydroxyl groups and other functional groups in their structures. Polysaccharides, including chitosan, alginate pectin, starch, chitin, carrageenan and cellulose derivatives, are commonly utilized for the synthesis of hydrogels (91). The formed hydrogels based on one or more kinds of polysaccharides can be applied to achieve the targeted delivery of bioactive molecules to special site. For example, alginate with the structures of rich anionic groups can interact with metal cations such as calcium ions to fabricate hydrogels which are widely studied as carriers to assist in delivering bioactive substances (87).

Remarkably, the combination of protein and polysaccharide can promote to synthesize the composite hydrogels with richer gelling behaviors than single component hydrogel, and thus showing prospective potential in exchanging nutrients (92). Moreover, the functional properties and digestibility of protein can be tunable when the blends composed of protein and polysaccharide are the source to form composite hydrogels. For instance, a study was conducted on the properties and functionalities of egg white protein heat-induced hydrogels with the addition of gellan gum, showing that the composite hydrogels had the appealing advantages of improved swelling behavior, enhanced water holding capacity, and tighter microstructures than the corresponding egg white protein hydrogels without the presence of gellan gum (93). More importantly, this study revealed that the composite hydrogels exhibited significantly increased stability when exposed to *in vitro* GI systems (94).

### Targeted delivery of hydrogels in the gastrointestinal tract

The most important capability of hydrogels in delivery systems is to release the loading substances at a specific location, which is very challenging for hydrogels design. In the situations where hydrogels cannot be placed directly in the body, especially in the locations with fluid, chemical and mechanical dynamic environments, prolonged gelation time and poor adhesion to the target location after gelation can lead to loss of hydrogel volume and embedded material, thus affecting the therapeutic effect (94).

Hydrogels suitable for release at low pH are relatively rare. Black carrot concentrate embedded in hydrogels made from whey protein isolate and pectin was not released slowly, instead it was promoted to release due to the structural defects of the gel which provided a more permeable environment for the passage of gastric fluid in and out (95). This hydrogel could be suitable for acidic food systems that should quickly be consumed (95).

Hydrogels delivered to stomach often require strong adhesion and acid stability, which presents a challenge for the design of hydrogels. In clinical practice, for the treatment of gastric bleeding, a hydrogel based on Poly(γ-glutamic acid) and tannic acid (96) was designed. This hydrogel has strong adhesion under wet conditions, strong tensile properties that can withstand long-term mechanical forces, good biocompatibility and safety in clinical trials (96). Tough hydrogels with trigger properties can also withstand harsh acidic conditions in the stomach when ingested, and can change shape or expand to ensure long-term retention and mechanical integrity, resolving side effects by triggering dissolution (97). *In situ* hydrogels can be used in the body once the hydrogels can form quickly and they are highly adhesive and can form a protective barrier on the surface where the drug can be released slowly (94, 98). Although *in situ* hydrogel formation is also a good strategy to target the stomach, there are still operational challenges for the delivery of food bioactive substances, unlike endoscopy in clinical practice.

Hydrogel delivery systems mostly target the intestine. In order to make a food bioactive substance arrive at the intestinal tract, it must withstand the acidic environment of the stomach and then be released slowly in the intestinal tract at a reasonable concentration. The pH-responsive hydrogels have attracted much attention in the application of intestinal targeting delivery. Chitosan is commonly used to prepare the pH-responsive hydrogels, which is degraded by the microflora in the colon and remains undigested in the upper gastrointestinal tract. However, hydrogels prepared from pure chitosan are highly porous and fragile, which are generally tend to absorb water and inefficient in drug delivery (99). The pH-responsive hydrogels composed of chitosan and glutaraldehyde showed higher swelling in neutral medium than in acidic medium, and the addition of β-cyclodextrin prolonged the drug release duration (100). The pH-responsive hydrogels sometimes have enzyme-catalyzed degradation (101, 102), and assembly materials of hydrogels are designed by targeting enzymes that need to respond. Hydrogels synthesized with biodegradable oligopeptides as crosslinking agents exhibit pH-responsive swelling and enzyme-catalyzed degradation, targeting pancreatic enzymes present in the small intestine (101). Hydrogels that respond to amylase are made of methacrylic acid and carboxymethyl starch (102). A novel hydrogel formed by a polymer in gastric fluid can protect the encased material from enzymatic degradation (103). In intestinal fluid, hydrogels can also inhibit the activity of proteases by depriving the enzyme of calcium ions, thus preventing the degradation of the encapsulated material (103).

**Figure 5:**
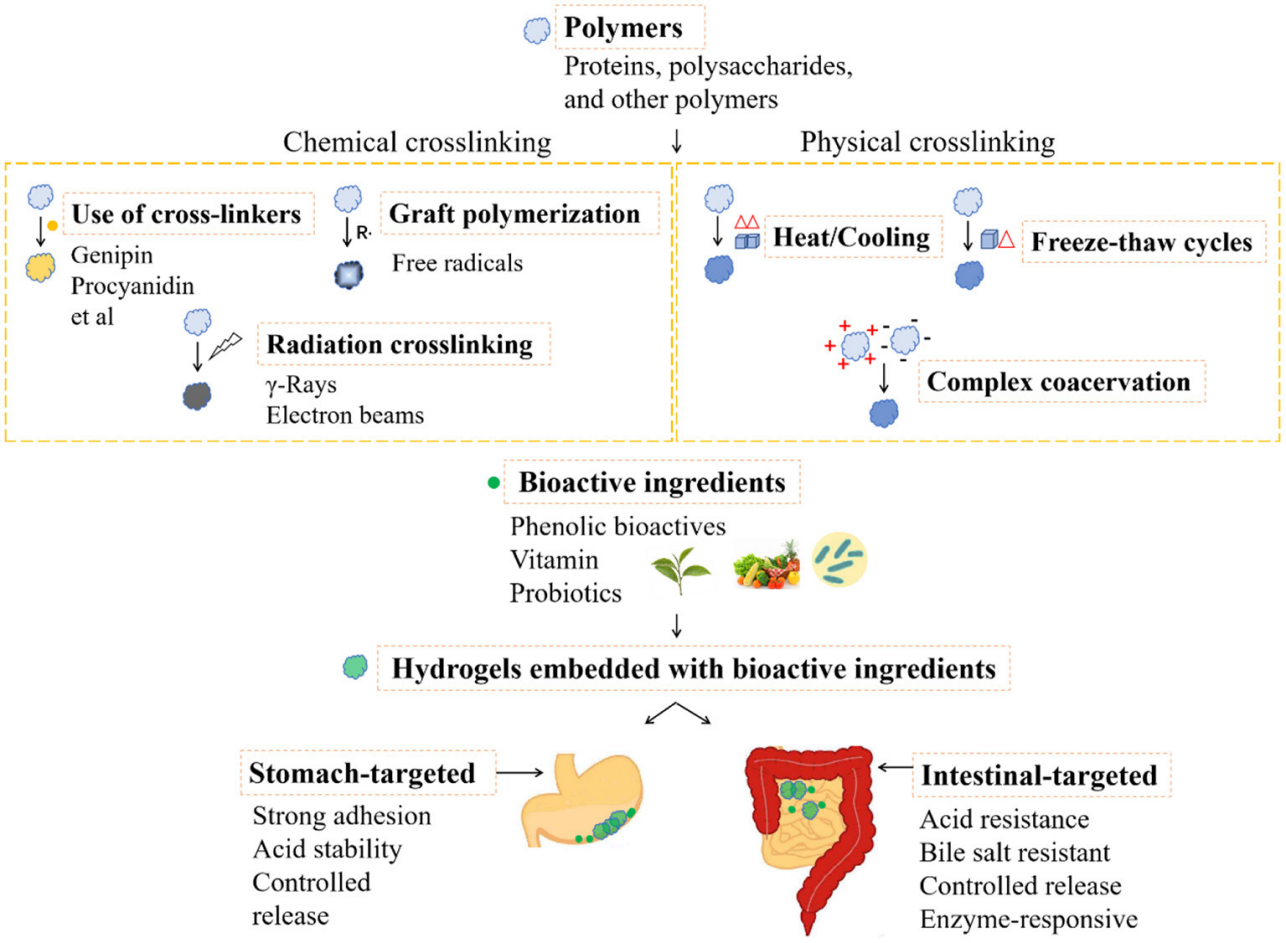
The preparation and delivery of the food functional ingredients by hydrogels for oral administration.

With the applications in food, hydrogels composed with edible biomacromolecules are commonly safe and popular. In food research field, it prefers the utilization of physical crosslinking in hydrogel preparation. Safety should be taken into consideration before the utilization of chemical crosslinking method. For example, the application of food grade crosslinkers, controlled free radical based grafting, and the physical field induced radiation crosslinking have the potentials in development of food hydrogels, which however are still in infant. Due to the harsh environmental conditions in GI tract, the hydrogels for delivery of nutraceuticals are expected to have the properties of strong adhesion, acid stability, resistance to bile salts and controlled release. On the other hand, the different pH conditions, digestive enzymes, as well as the proteases secreted by gut microbiota could be taken as the stimulations for design of the environmental trigger hydrogels. Here, the phenolic bioactives, vitamins and probiotics are taken for the main instances as the nutraceuticals embedded by the hydrogels in oral administration. The preparation and delivery of the food functional ingredients by hydrogels for oral administration are briefly summarized in Figure 5.

### Delivery of phenolic bioactives

**Figure 6:**
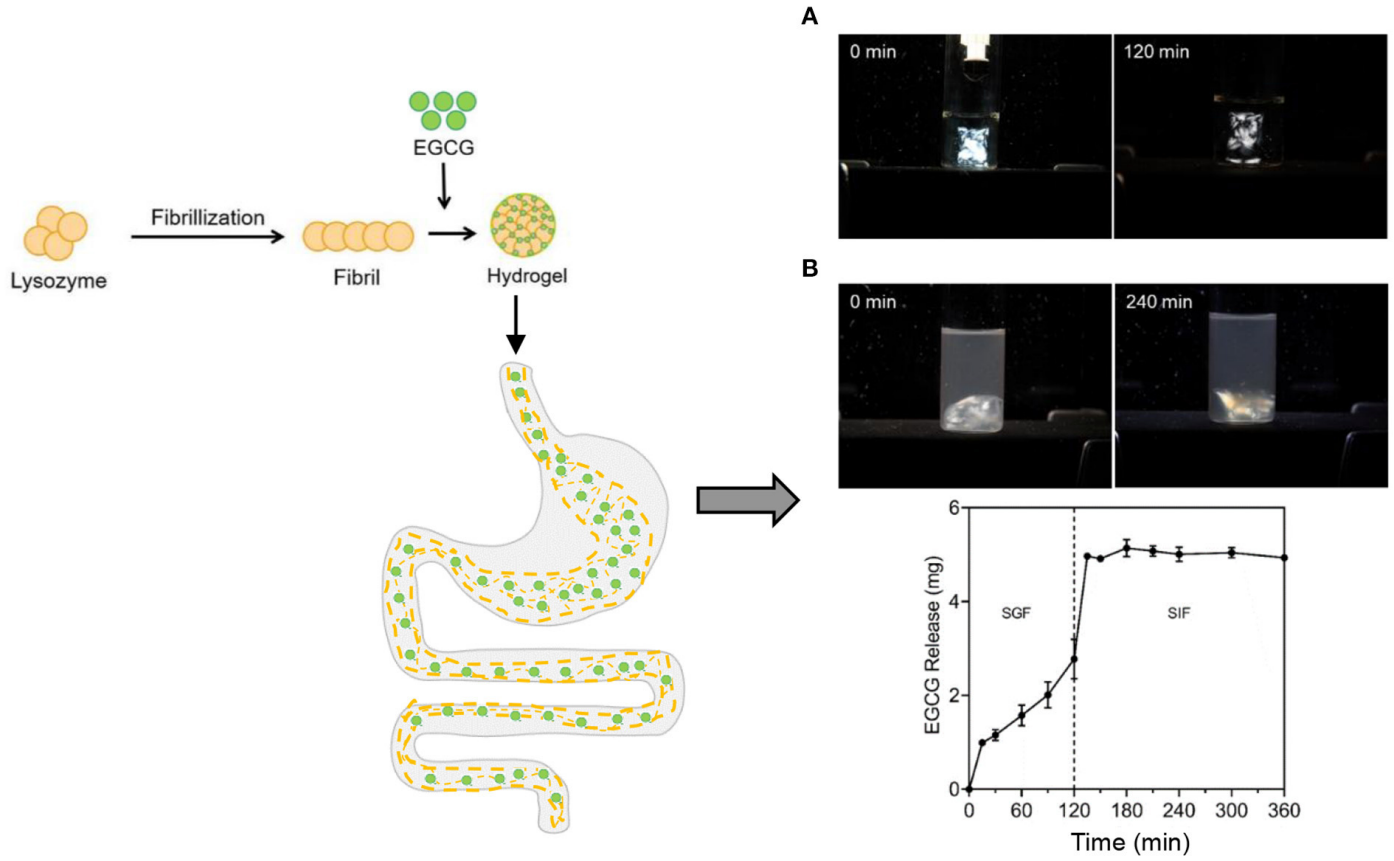
The formation of cold-set hydrogels formed through the self-assembly of polyphenol epigallocatechin gallate (EGCG) and the protein fibrils originating from egg white lysozyme as well as the digestion of the hydrogels and the controlled release of the encapsulated EGCG in the simulated GI tract: **(A)** simulated gastric fluid (SGF) and **(B)** simulated intestinal fluid (SIF). The hydrogels showed birefringence under the polarized light.

Phenolic bioactives are widely abundant in nature such as curcumin, quercetin, and epigallocatechin-3-gallate (EGCG), which are known for their attractive biological properties like anti-inflammatory activity. However, they are commonly sensitive to light, heat, and other harmful conditions, and thus having poor stability (104). Numerous studies have been conducted to develop the hydrogels incorporated with phenolic compounds as delivery systems to improve the stability and even realize the sustained release. The cold-set hydrogels formed through the self-assembly of polyphenols and the protein fibrils originating from egg white were developed, which were demonstrated to perform largely increased loading of polyphenols in contrast with other carriers, and showed resistance to the digestion in stomach whereas showed controlled release in intestine (Figure 6) to achieve targeted treatment of intestinal inflammation *via* drugging the gut microbiota (105–107). More than 16 different kinds of polyphenols were found to co-assemble with the protein fibrils to form the hydrogels (105–107). Specifically, oral administration of the polyphenol-protein fibril hydrogels reduced the abundances of normally enriched microorganisms highly related to colitis, especially targeting facultative anaerobes of the phylum Proteobacteria, such as *Aestuariispira* and *Escherichia* (106). Furthermore the polyphenol-protein fibril hybrid hydrogels were recently found to prevent the high-fat diet induced obesity through construction of gut microbiota (107). The gut microbiota dysbiosis caused by high fat diet (HFD) induced obesity was markedly ameliorated. Overexpression of the host intestinal lipid absorption genes *CD36* and *NFIL3* decreased significantly. Furthermore, transplantation of the gut microbiota educated by the hydrogels to germ-free mice showed substantial prevention effect on HFD-induced obesity, accompanied by a distinct microbiota structure that resisted the HFD-induced divergence in microbiota structure (107).

In addition, two or more components of bioploymers are often used to obtain the hydrogels to seek the preferred properties by adjusting the proportion of each component. Novel polyelectrolyte complex hydrogels were developed by self-assembly of two kinds of polysaccharides including salecan and N,N,N-trimethyl chitosan (TMC) for delivering the embedded green tea polyphenols, and the properties of hydrogels and *in vitro* release behavior of polyphenols were studied (68). It was found that the polyphenols had obvious higher release in simulated intestinal fluid compared with simulated gastric fluids, and the increased release of polyphenols can be obtained with the increased ratio of salecan/TMC (68). On the other hand, for the hydrophobic phenolic bioactives like curcumin, they can also be embedded into hydrogels to build delivery systems. Curcumin has been reported to possess many functional properties, such as antimicrobial, anticancer and anti-inflammatory activity (108), but they are limited to low solubility and unsatisfied delivery, and thus it is necessary to improve these disadvantages to exert their functionalities by encapsulation. For instance, novel nanogels prepared with acylated rapeseed protein isolate were produced by acylation and heat-induced protein, which possessed the increased hydrophobic structures and were used to encapsulate hydrophobic curcumin, showing the outstanding encapsulation efficiency up to 95% and the effective resistance to wide range of pH and ionic strength (109). And the release of the encapsulated hydrophobic curcumin could mainly depend on the erosion and breakup of nanogels. In another recent study, the cold-set mixed hydrogels comprised of whey protein aggregates and κ-carrageenan were used to encapsulate curcumin, and the encapsulation efficiency and the release in simulated gastrointestinal tract of curcumin were evaluated (110). The result revealed that the higher content of curcumin was incorporated into the mixed gels than the single component hydrogel without the addition of κ-carrageenan, and showed that when the mixed hydrogels were delivered to the simulated digestive systems, only a few parts of loaded curcumin degraded in gastrointestinal digestion whereas more than 87% of loaded curcumin was released in colon due to the beneficial protection of κ-carrageenan (110). For quercetin, also a hydrophobic molecule, Hu et al. encapsulated the quercetin dissolved in corn oil with the mixture of sodium alginate and the conjugates of EGCG modified soy protein isolate to prepare hydrogel microspheres, which maintained good morphology during digestion (111). And only part of quercetin was digested in the upper gastrointestinal tract. Lipogels prepared by embedding of liposomes loading with quercetin into the hydrogels consisted of gelatin and chitosan showed a significant protective effect on the gastric release of quercetin (112). In addition, the strength of the lipogels could be changed by adjusting the ratio of gelatin to chitosan, and the soft lipogels released more quercetin during gastrointestinal digestion (112).

### Delivery of vitamin

Vitamins are important compounds to promote human health, mainly classified into hydrophilic and lipophilic vitamins. Hydrophilic vitamins mainly include vitamin C and a class of vitamin B. It is necessary to develop the encapsulation system to resist the effect of adverse conditions on the stability of vitamins. For example, polyelectrolyte complex hydrogels loaded with vitamin C were self-assembled from natural polysaccharides salecan and chitosan with opposite charges, and *in vitro* release curves showed pH-triggered and sustained release characteristics (113). The result showed that the release of vitamin C in the simulated gastric fluid was very limited, while the release amount of vitamin C reached 92.3% once entering the simulated intestinal fluid (113). Whey protein-based hydrogel microbeads incorporated with riboflavin were prepared by the process of cold-set gelation and drying technique to evaluate the release *in vivo* gastrointestinal tract, and the result showed that riboflavin was difficult to degrade in the stomach and small intestine, and it could be released to exert effective function after reaching the colon (114). The hydrogels based on the alginate in combination with the divalent copper were studied to realize the encapsulation for folic acid and further to explore their stability in the simulated physiological pH conditions, and the result showed that the hydrogels were able to resist the effect of acidic pH while realizing the sustained release at simulated intestinal conditions (115). Also, the hydrogels based on the diverse composition of alginate and pectin were developed to encapsulate folic acid to seek the favorable protection, and the result showed that the composite hydrogels provided the stronger protective effect than the hydrogels without the coating of pectin when they are exposed to the simulated gastric conditions, and the sustained release behavior of folic acid was observed in simulated intestinal conditions (116). On the other hand, lipophilic vitamins mainly consist of a class of vitamins A, D, E, and K. These fat-soluble vitamins are encapsulated in food hydrophilic colloids and can be targeted for delivery to specific sites in the gastrointestinal tract or regulator release. Loading vitamin D3 into a composite gel made from whey protein isolate and lotus root amylopectin can protect it from photodegradation and thus improve storage stability (117). In addition, the gel can improve the bioavailability of vitamin D3 (117). A study developed salt-induced protein gels based κ-lactoglobulin or hen egg white protein to encapsulate κ-tocopherol, and the result showed that most of κ-tocopherol were observed to release in simulated gastric conditions (118). When κ-lactoglobulin protein-based gels coated with alginate, about 55% of κ-tocopherol could be retained to release in simulated intestinal conditions (118).

### Delivery of probiotics

Probiotics are dietary supplements of living microbial that are believed to play an important role in maintaining health because of multiple beneficial effects on the host system when consumed in moderate amounts (119). According to the Food and Agriculture Organization-World Health Organization (FAO-WHO), the minimum level of probiotics should be 10^7^ CFU/g to achieve desired therapeutic effects (120, 121). In the process of passing through the gastrointestinal tract, probiotics not only suffer from the catalytic action of gastric enzymes and the harsh acidic environment of the stomach, but also resist the antibacterial activity of intestinal bile salts and the action of protease, which puts forward high requirements for probiotics to reach the intestinal tract in a certain number (122, 123). Hydrogels can provide the protection for probiotics, and compared with the hydrogels sourced from single component, hydrogels with multicomponent materials as carrier of probiotics can enable higher concentrations of probiotics to reach specific locations under the same initial concentration, which means it can provide better protection for probiotics (124–126). The survival rate of *Lactobacillus plantarum* ATCC: 13,643 in pectin/starch hydrogels was significantly higher than that of free cells in simulated gastric fluid and bile salt solution, indicating that hydrogels can resist the adverse conditions of gastrointestinal and bile salt solution and enable probiotics to be delivered to the colon (119). Data showed that after sequential exposure to simulated gastric fluid and simulated intestinal fluid for 2 h almost complete death of free cells was observed, however the numbers of surviving cells were still 10^5^ to 10^6^ CFU/g for hydrogels of different compositions of pectin alone and pectin/starch mixture (119). In addition, hydrogels are designed to be pH-triggered so that they remain structurally stable in acidic environments, but gradually disintegrate in neutral pH environments, thus releasing probiotics in the intestinal tract (127). The hydrogels designed by Wang et al. (127), based on sodium carboxymethyl cellulose/chitosan and sodium alginate/calcium chloride systems, can maintain a reticular shell structure for 3 h in simulated gastric fluid and begin to decompose in intestinal fluid for 2 h, with a sustained release duration of more than 10 h.

It has been reported that probiotics have been successfully encapsulated in water-in-water (W/W) and water-in-oil-in-water (W/O/W) emulsions (128). However, probiotics often come into direct contact with the water phase in the emulsions, resulting in probiotics inactivation. Gao et al. (129) used whey protein isolate and pectin to prepare oil-in-water high internal phase emulsion, and added D-gluconate-δ-lactone and calcium to obtain a double-network high internal phase emulsion gels. High internal phase emulsions (HIPEs) are a unique type of emulsion with internal phase fraction over the highest geometric limit of the rigid closely packed spheres, 74%, and therefore droplets are highly packed and deformed into polyhedral geometries. *In vitro* digestibility showed that the high internal phase emulsion with pectin inhibited the contact of probiotics with gastric acid and bile salts, because of the formation of a double-network gel structure and tight oil droplet extrusion, enhancing the activity of probiotics in the gastrointestinal tract (129).

In terms of drug delivery, hydrogel-loaded probiotics have a more specific orientation. The novel γ-glutamate hydrogel microcapsule will rapidly release probiotics when response to nitric oxide stimulation to maintain the integrity of the intestinal barrier and regulate the balance of gut microbiota, thus improving the therapeutic effect of sodium glucan sulfate induced colitis (130). In this study, the generation of NO caused by intestinal inflammation was utilized as a stimuli to trigger the breakup of the γ-glutamate hydrogel microcapsule, to release the encapsulated probiotics. Similar to gastric adhesion (131, 132), colon-targeted adhesion delivery system reduces systemic exposure time and prolongs local drug retention time (133). Interestingly, it has been found that gene editing allows probiotics like clinical *Bacillus subtilis, E. coli* Nissle 1917 to be self-coated by the secreted curli nanofibers, the major protein in biofilm, to form the living proteinaceous hydrogels or biofilms which in turn protect themselves from the acidic environment of the stomach and attach to the small intestine (134–136). The primary structural component of curli nanofibers is the CsgA protein, whose cell-directed assembly can be programmed *via* gene editing. Furthermore, CsgA could be genetically fused to a therapeutic domain proteinaceous ingredients, such as cytokine secreted by mucus-producing cells, to endow the living hydrogels with stronger mucosal healing functions. This provides a new approach for the delivery of probiotics in food for reference. Besides performing as the encapsulation and delivery systems, biopolymer, such as cellulose hydrogels have been developed to construct the gut-like bioreactors for growth of multiple-strain probiotic bacteria (137).

The details about the encapsulation, delivery and function of different nutraceuticals by hydrogels are listed in the following Table 1.

**Table 1:** The preparation method of different hydrogels for encapsulation and delivery of nutraceuticals including phenolic bioactives, vitamins and probiotics, as well as their functions.

**The nutraceuticals**	**Hydrogels**	**The preparation methods**	**Functions**
Polyphenols (106)	egg white lysozyme hydrogels	The purified lysozyme monomers were lyophilized, which were further heated in pH 2 solution with protein concentration of 2 wt% in 90 °C oil bath for 8 hours under agitation, to fabricate the amyloid fibrils. Polyphenols were dissolved respectively in the 10 mMBis-Tris buffer (pH 6.8), The 2 wt% amyloid fibril solution (pH2) was blended with the polyphenol solution in equal volume for preparation hydrogels.	Hydrogels significantly promoted intestinal barrier function, suppressed the proinflammatory mRNA expression, and very significantly regulated gut microbial dysbiosis.
Green tea polyphenols (68)	novel polyelectrolyte complex hydrogels were developed by self-assembly of two kinds of polysaccharides including salecan and N,N,N-trimethyl chitosan	The salecan and TMC solutions were prepared, respectively. Salecan solution was added dropwise into TMC solution according to various volume ratios under sonication for 30min. Self-assembly precursor solution was thus formed and then poured into a circular glass mold at room temperature. The molds were then placed in a desiccator containing an appropriate amount of acetic acid solution and stayed for 2h until the solutions were transformed into PEC hydrogels.	The PEC hydrogels could play a good role of intestinal targeted nutrition transport.
Hydrophobic curcumin (109)	self-assembled acylated rapeseed protein isolate nanogels	A new biocompatible and self-assembled acylated rapeseed protein isolate(ARPI) based nanogels were fabricated by the chemcial acylation and heat-induced protein denaturation. Protein acylation reaction was performed on RPI with butanedioic anhydride.	Significantly increasing its anticancer activity against multiple cancer cell lines
Curcumin (110)	mixed hydrogels composed of whey protein aggregates (WPA)/k- carrageenan	Preparation of whey protein aggregates (WPA) by free radical cross-linking method. Curcumin dissolved in ethanol was mixed in a certain proportion with the prepared WPA solution for 8h at room temperature for prepare curcumin-loaded whey protein aggregates. Carrageenan prepared with polysaccharide hydrated overnight at 25°C was added to the WPA solution for preparation of polysaccharide/protein mixed gel pre-solutions.The gel pre-solutions were charged by 1.3% w/v GDL for formation GDL-induced gel.	Protecting curcumin within the upper gastrointestinal tract and deliver it to the colon.
Quercetin (111)	gelatin-chitosan hydrogels	Based on the changes in functional groups in the FTIR and DSC heatmaps, quercetin-loaded liposomes were embedded in a gelatin-chitosan hydrogel and ionic and covalent bonds between Na^+^ and mTGase reactions.	This lipid gel system can track multifunctional and effective molecules by changing their structural properties for controlled release in specific pH or enzyme induced burst environments.
Vitamin C (113)	salecan/chitosan PEC hydrogels	Chitosan was added to the salecan solution and mixed,and PEC hydrogels were formed upon exposure to acetic acid atmosphere for 3h.VC was loaded onto the PEC hydrogels using an equilibrium partitioning method.	Hydrogels showed excellent cytocompatibility and biodegradability.It can show a good nutrient delivery function in specific parts of the intestine.
Riboflavin (114)	whey microbeads	Microbeads was prepared by dissolving the denaturing whey protein solution by Gilson minipuls in CaCl_2_ for several hours.	Drying of the microbeads provided a significant decrease in riboflavin release rate *in vitro* compared to wet microbeads and also impeded microbead degradation.
Folic acid (115)	folic acid-copper alginate hydrogels	Folic acid hydrogels were prepared by mixing 1.3%(w/v) sodium alginate and 50%(w/w, wrt sodium alginate) folic acid for 20 minutes. The folic acid-sodium alginate solution and was added dropwise to the CuSO4 solution, and folic acid-copper alginate gels were formed in the process.	The copper alginate acted as gastro-resistant material and slow release of folic acid occurs.
Folic acid (116)	the compositions of biocomposite consisted of alginate and pectin	Alginate and pectin were mixed and dissolved in CaCl_2_ by a syringe pump to prepare into blank microglue, and folic acid was dispersed in this composite microcapsule.	Composite hydrogels provided the stronger protective effect and the sustained release behavior of folic acid was observed in simulated intestinal conditions.
Vitamin D3 (117)	the composite gel of whey protein isolate and lotus root amylopectin(WPI-LRA gels)	LRA and WPI solutions were prepared, mixed and heated through a water bath into the gels.Vitamin D3 were dissolved in ethanol,then was added to the WPI-LRA mixture,finally The above mixtures are heated,cooled and lyophilized.	This encapsulation could increase the storage stability of vitamin D3 and protect vitamin D3 from photochemical degradation. The *in vitro* experiment suggested that WPI-LRA composite gel could supply a protective barrier for vitamin D3 and prolong the residence time in intestine.
α-tocopherol (α-TOC) (118)	salt-induced proteingels based β-lactoglobulin or hen egg white protein	Salt-induced gelation technique was used for preparation of protein based-encapsulated α-TOC. Appropriate concentration of α-TOC was mixed with appropriate concentration of BLG solution and subsequently CaCl_2_, was added to induce aggregation of BLG. A method to prepare HEW-encapsulated α-TOC was similar to that of the BLG-encapsulated α-TOC except that ZnCl_2_ was added instead of CaCl_2_ to induce aggregation of BLG.	With the alginate coat, the release of α-TOC was retarded till intestinal stage and the encapsulation efficiencies of α-TOC by BLG and HEW were enhanced.
Lactobacillus plantarum ATCC:13643 (119)	pectin/starch hydrogels	Pectin/starch hydrogel were prepared by external gelation method at various pectin/starch ratios.Lactobacillus plantarum ATCC:13643 cells were encapsulated in pectin/starch hydrogel by extrusion method.	Incorporation of starch with pectin biopolymer provided significant protection for cells against the harsh conditions of simulated gastric tract.The pectin/starch hydrogel increased the tolerance of L. plantarum to strongly acidic media and bile solutions and enable probiotics to be delivered to the colon.
Probiotic Lactobacillus reuteri (128)	heteroprotein complex coacervation (type-A gelatin/sodium caseinate, GE/Cas)	The microcapsules were prepared using the method of coacervation or mixing, followed by spray drying.The control GE and Cas microcapsules were prepared at pH 6.0. The operation temperature for coacervation was kept at 40°C and the biopolymer solutions, sucrose and the probiotics were mixed.The mixture was finally adjusted with ph, ice bath and drying treatment.	Microencapsulation in GE/Cas improved the survival during dry storage,and the stability of the probiotic cells was improved.
Probiotics (130)	A novel NO-responsivepoly-γ-glutamic acid(γ-PGA) hydrogel microcapsule (NRPM)	Using a visible light poly-merization method to produced macroscopic NRPM hydrogels. The gelation mechanism was attributed to the formation of covalent C-C linkages between the C=C groups on the γ-PGA-GMA backbone and the terminal of	Owing to the cytoprotective effects of the NRPM, the decorated probiotics showed high viability in the simulatedgastric and intestinal fluid
		the APD.Meanwhile, it retained a benzotriazole group that acted as a targeted molecule capable of responding to NO. A transparent and homogeneous hydrogel formed within a controllable time from several seconds to several minutes.	environments. Microspheres can respond to nitric oxide(NO) stimuli and rapidly release probiotics to maintain the intestinal mechanical barrier and regulate the balance of intestinal flora. NRPM is a promising approach for improving the efficacy of orally administered probiotics in patients with colonic IBD.
Bacillus subtilis (BS) (135)	Self-coating with BS biofilms	Firstly, the biofilm-free BS was obtained, then the seed medium was suspended to obtain FCBS, and the cell pellets were collected and then suspended in PBS, the resulted solution was spread on solid MSgg plates. Robust BS biofilms were produced after 2 days of culture at 30°C. Individually coated bacteria BCBS were prepared by homogenizing the films with PBS.	Self-coating with biofilms that endows the transplanted gut microbiota with superior resistance and adhesion capacity.Coated probiotics exhibit a higher oral bioavailability,intestinal colonization and notable ability to survive and reside in the GI tract.

## Conclusion and challenge

In comparison with other carries with the size in microscopic scale such as nanoparticle and microcapsules, hydrogels are expected to be more suitable delivery systems in food due to their macroscopic bulk properties, adjustable viscoelasticity and large spatial structure for embedding nutraceuticals. Despite the generally lower strength and toughness than the synthetic polymers, natural polymer-based hydrogels are commonly safe and popular in development of delivery systems in food. And it prefers the physical crosslinking approaches in the fields of food, which have the advantages of safety, reversibility and feasibility of realizing environmental stimuli properties. The reasonable design for the structure of natural polymeric hydrogels is essential for seeking the favorable functionalities to apply in the delivery system, because it is possible to obtain the enhanced stability and the controlled release behavior of the embedded bioactive ingredients. The design of environmental stimuli hydrogels has taken the different pH conditions, digestive enzymes, as well as the metabolism of gut microbiota as the stimulations. Targeted delivery of hydrogels in GI tract has shown some successful cases, which however is still in infant. The hydrogels prepared with proteins, polysaccharides or the mix of them to deliver the functional ingredients, mainly the phenolic components, vitamins, probiotics are discussed to obtain inspiration for the wide applications in delivery systems. The *in situ* formation of hydrogels through the interaction among food biomacromolecules and small-molecular ingredients could deserve further investigations and efforts. In order to exert the bioactivities of the loading nutraceuticals, their loading contents in hydrogels have to be enhanced. Utility of stomach-targeted hydrogels for delivery of bioactive food ingredients should be investigated for the prevention or treatment of stomach ulcer and the eradication of *Helicobacter pylori*. Directional transport to colon for targeting of gut microbiota should be essential for realization of the health benefits of future food hydrogels.

## Author contributions

ML, XH, RZ, and QS: writing—original draft. YN and BH: writing—review and editing, supervision, project administration, and resources. All authors approved the final manuscript.

## Funding

Laboratory of Lingnan Modern Agriculture Project (NZ2021034); the National Natural Science Foundation of China—Youth Foundation (No. 31901760); the Natural Science Foundation of Jiangsu Province—Distinguished Youth Foundation (BK20200022); the National Natural Science Foundation of China (No. 31871843); the Natural Science Foundation of Jiangsu Province—Youth Foundation (BK20190530); Jiangsu Agricultural Science and Technology Innovation Fund [CX(21)3040].

## Conflict of interest

The authors declare that the research was conducted in the absence of any commercial or financial relationships that could be construed as a potential conflict of interest.

## Publisher's note

All claims expressed in this article are solely those of the authors and do not necessarily represent those of their affiliated organizations, or those of the publisher, the editors and the reviewers. Any product that may be evaluated in this article, or claim that may be made by its manufacturer, is not guaranteed or endorsed by the publisher.

## References

[1] Mozaffarian D. Dietary and policy priorities for cardiovascular disease, diabetes, and obesity a comprehensive review. Circulation. (2016) 133:187–225. 10.1161/CIRCULATIONAHA.115.018585 26746178 PMC4814348

[2] Mozaffarian D. Dietary and policy priorities to reduce the global crises of obesity and diabetes. Nat Food. (2020) 1:38–50. 10.1038/s43016-019-0013-1

[3] Pan MH Ho CT. Chemopreventive effects of natural dietary compounds on cancer development. Chem Soc Rev. (2008) 37:2558–74. 10.1039/b801558a 18949126

[4] Yang CS Wang X Lu G Picinich SC. Cancer prevention by tea: animal studies, molecular mechanisms and human relevance. Nat Rev Cancer. (2009) 9:429–39. 10.1038/nrc2641 19472429 PMC2829848

[5] Assadpou E Jafari SM. A systematic review on nanoencapsulation of food bioactive ingredients and nutraceuticals by various nanocarriers. Crit Rev Food Sci Nutr. (2018):1–47. 10.1080/10408398.2018.1484687 29883187

[6] Bing H Minhao X Chen Z Xiaoxiong Z. Genipin-structured peptide–polysaccharide nanoparticles with significantly improved resistance to harsh gastrointestinal environments and their potential for oral delivery of polyphenols. J Agric Food Chem. (2014) 62:12443–52. 10.1021/jf5046766 25479066

[7] Bing H Xixia L Chunlan Z Xiaoxiong Z. Food macromolecule based nano-delivery systems for enhancing the bioavailability of polyphenols. J Food Drug Anal. (2017) 25:3–15. 10.1016/j.jfda.2016.11.004 28911541 PMC9333428

[8] Ahmed EM. Hydrogel: preparation, characterization, and applications: a review. J Adv Res. (2015) 6:105–21. 10.1016/j.jare.2013.07.006 25750745 PMC4348459

[9] Ullah F Othman MBH Javed F Ahmad Z Akil HM. Classification, processing and application of hydrogels: a review. Mater Sci Eng C Mater Biol Appl. (2015) 57:414–33. 10.1016/j.msec.2015.07.053 26354282

[10] Dickinson E. Microgels - an alternative colloidal ingredient for stabilization of food emulsions. Trends Food Sci Tech. (2015) 43:178–88. 10.1016/j.tifs.2015.02.006

[11] Renard D van de Velde F Visschers RW. The gap between food gel structure, texture and perception. Food Hydrocolloid. (2006) 20:423–31. 10.1016/j.foodhyd.2005.10.014

[12] McClements DJ. Designing biopolymer microgels to encapsulate, protect and deliver bioactive components: physicochemical aspects. Adv Colloid Interface Sci. (2017) 240:31–59. 10.1016/j.cis.2016.12.005 28034309

[13] Micale N Citarella A Molonia MS Speciale A Cimino F Saija A. Hydrogels for the delivery of plant-derived (Poly)phenols. Molecules. (2020) 25:3254. 10.3390/molecules25143254 32708833 PMC7397257

[14] McClements DJ. Recent progress in hydrogel delivery systems for improving nutraceutical bioavailability. Food Hydrocoll. (2017) 68:238–45. 10.1016/j.foodhyd.2016.05.037

[15] Zhao W Jin X Cong Y Liu YY Fu J. Degradable natural polymer hydrogels for articular cartilage tissue engineering. J Chem Technol Biot. (2013) 88:327–39. 10.1002/jctb.3970

[16] Mahinroosta M Farsangi ZJ Allahverdi A Shakoori Z. Hydrogels as intelligent materials: a brief review of synthesis, properties and applications. Mater Today Chem. (2018) 8:42–55. 10.1016/j.mtchem.2018.02.004

[17] Madduma-Bandarage USK Madihally SV. Synthetic hydrogels: synthesis, novel trends, and applications. J Appl Polym Sci. (2021) 138:50376. 10.1002/app.50376

[18] Liu LS Kost J Yan F Spiro RC. Hydrogels from biopolymer hybrid for biomedical, food, and functional food applications. Polymer. (2012) 4:997–1011. 10.3390/polym4020997

[19] Islam A Yasin T Bano I Riaz M. Controlled release of aspirin from pH-sensitive chitosan/poly(vinyl alcohol) hydrogel. J Appl Polym Sci. (2012) 124:4184–92.

[20] Hu WK Wang ZJ Xiao Y Zhang SM Wang JL. A dvances in crosslinking strategies of biomedical hydrogels. Biomater Sci. (2019) 7:843–55. 10.1039/C8BM01246F 30648168

[21] Koetting MC Peters JT Steichen SD Peppas NA. Stimulus-responsive hydrogels: theory, modern advances, and applications. Mater Sci Eng R Rep. (2015) 93:1–49. 10.1016/j.mser.2015.04.001 27134415 PMC4847551

[22] Deng ZX Yu R Guo BL. Stimuli-responsive conductive hydrogels: design, properties, and applications. Mater Chem Front. (2021) 5:2092–123. 10.1039/D0QM00868K33406183

[23] Krogsgaard M Behrens MA Pedersen JS Birkedal H. Self-healing mussel-inspired multi-pH-responsive hydrogels. Biomacromolecules. (2013) 14:297–301. 10.1021/bm301844u 23347052

[24] Doering A Birnbaum W Kuckling D. Responsive hydrogels - structurally and dimensionally optimized smart frameworks for applications in catalysis, micro-system technology and material science. Chem Soc Rev. (2013) 42:7391–420. 10.1039/c3cs60031a 23677178

[25] Wu Lihuang LiW. Cai Xiaojun. Research progress of stimulus-responsive hydrogels for controlled drug delivery. Chinese J Bioproc Eng. (2020) 18:806–14. 10.3969/j.issn.1672-3678.2020.06.017

[26] Culver HR Clegg JR Peppas NA. Analyte-responsive hydrogels: intelligent materials for biosensing and drug delivery. Acc Chem Res. (2017) 50:170–8. 10.1021/acs.accounts.6b00533 28170227 PMC6130197

[27] Li JL Jia X Yin LJ. *Hydrogel:* diversity of structures and applications in food science. Food Rev Int. (2021) 37:313–72. 10.1080/87559129.2020.1858313

[28] Oh JK Lee DI Park JM. Biopolymer-based microgels/nanogels for drug delivery applications. Prog Polym Sci. (2009) 34:1261–82. 10.1016/j.progpolymsci.2009.08.001

[29] Torchilin VP. Multifunctional, stimuli-sensitive nanoparticulate systems for drug delivery. Nat Rev Drug Discov. (2014) 13:813–27. 10.1038/nrd4333 25287120 PMC4489143

[30] Kabanov AV Vinogradov SV. Nanogels as pharmaceutical carriers: finite networks of infinite capabilities. Angew Chem Int Ed Engl. (2009) 48:5418–29. 10.1002/anie.200900441 19562807 PMC2872506

[31] Batista RA Espitia PJP Quintans JDS Freitas MM Cerqueira MA Teixeira JA. Hydrogel as an alternative structure for food packaging systems. Carbohydr Polym. (2019) 205:106–16. 10.1016/j.carbpol.2018.10.006 30446085

[32] Dash M Chiellini F Ottenbrite RM Chiellini E. Chitosan-A versatile semi-synthetic polymer in biomedical applications. Prog Polym Sci. (2011) 36:981–1014. 10.1016/j.progpolymsci.2011.02.001

[33] Hennink WE van Nostrum CF. Novel crosslinking methods to design hydrogels. Adv Drug Deliv Rev. (2002) 54:13–36. 10.1016/S0169-409X(01)00240-X 11755704

[34] Kosaraju SL Puvanenthiran A Lillford P. Naturally crosslinked gelatin gels with modified material properties. Food Res Int. (2010) 43:2385–9. 10.1016/j.foodres.2010.09.008

[35] Gao L Gan H Meng ZY Gu RL Wu ZN Zhang L. Effects of genipin cross-linking of chitosan hydrogels on cellular adhesion and viability. Colloids Surf B. (2014) 117:398–405. 10.1016/j.colsurfb.2014.03.002 24675278

[36] Vo NTN Huang L Lemos H Mellor AL Novakovic K. Genipin-crosslinked chitosan hydrogels: preliminary evaluation of the in vitro biocompatibility and biodegradation. J Appl Polym Sci. (2021) 138:e50848. 10.1002/app.50848

[37] Chen H Gan J Ji AG Song SL Yin LJ. Development of double network gels based on soy protein isolate and sugar beet pectin induced by thermal treatment and laccase catalysis. Food Chem. (2019) 292:188–96. 10.1016/j.foodchem.2019.04.059 31054664

[38] Gharibzahedi SMT Roohinejad S George S Barba FJ Greiner R Barbosa-Canovas GV. Innovative food processing technologies on the transglutaminase functionality in protein-based food products: trends, opportunities and drawbacks. Trends Food Sci Technol. (2018) 75:194–205. 10.1016/j.tifs.2018.03.014

[39] Liang YP Zhao X Ma PX Guo BL Du YP Han XZ. pH-responsive injectable hydrogels with mucosal adhesiveness based on chitosan-grafted-dihydrocaffeic acid and oxidized pullulan for localized drug delivery. J Colloid Interface Sci. (2019) 536:224–34. 10.1016/j.jcis.2018.10.056 30368094

[40] Li BG Zhang YD Wu C Guo B Luo ZY. Fabrication of mechanically tough and self-recoverable nanocomposite hydrogels from polyacrylamide grafted cellulose nanocrystal and poly (acrylic acid). Carbohydr Polym. (2018) 198:1–8. 10.1016/j.carbpol.2018.06.047 30092978

[41] Ma DZ Zhu BD Cao B Wang J Zhang JW. The microstructure and swelling properties of poly acrylic acid-acrylamide grafted starch hydrogels. J Macromol Sci B. (2016) 55:1124–33. 10.1080/00222348.2016.1242552

[42] Chandel AKS Nutan B Raval IH Jewrajka SK. Self-assembly of partially alkylated dextran-graft-poly (2-dimethylamino)ethyl methacrylate copolymer facilitating hydrophobic/hydrophilic drug delivery and improving conetwork hydrogel properties. Biomacromolecules. (2018) 19:1142–53. 10.1021/acs.biomac.8b00015 29486116

[43] Kim MH Park WH. Chemically cross-linked silk fibroin hydrogel with enhanced elastic properties, biodegradability, and biocompatibility. Int J Nanomedicine. (2016) 11:2967–78. 10.2147/IJN.S106467 27382283 PMC4922766

[44] Wu Nie Yu Huilei Sun Muyang Li Zong Zhao Fengyuan Ao Yingfang. Investigation on the structure and mechanical properties of highly tunable elastomeric silk fibroin hydrogels cross-linked by gamma-ray radiation. ACS Appl Energy Mater. (2020) 3:721–34. 10.1021/acsabm.9b01062 35019416

[45] Dehghan-Niri M Vasheghani-Farahani E Eslaminejad MP Tavakol M Bagheri F. Physicomechanical, rheological and in vitro cytocompatibility properties of the electron beam irradiated blend hydrogels of tyramine conjugated gum tragacanth and poly (vinyl alcohol). Mat Sci Eng C-Mater. (2020) 114:111073. 10.1016/j.msec.2020.111073 32994011

[46] Ajji Z Mirjaliji G Alkhatab A Dada H. Use of electron beam for the production of hydrogel dressings. Radiat Phys Chem. (2008) 77:200–2. 10.1016/j.radphyschem.2007.05.01621504052

[47] Raza MA Lim YM Lee SW Seralathan KK Park SH. Synthesis and characterization of hydrogels based on carboxymethyl chitosan and poly(vinylpyrrolidone) blends prepared by electron beam irradiation having anticancer efficacy, and applications as drug carrier for controlled release of drug. Carbohydr Polym. (2021) 258:117718. 10.1016/j.carbpol.2021.117718 33593580

[48] Susheel Kalia. Dehradun India. Polymeric Hydrogels as Smart Biomaterials. Springer International Publishing Switzerland. (2015).

[49] Croisier F Jerome C. Chitosan-based biomaterials for tissue engineering. Eur Polym J. (2013) 49:780–92. 10.1016/j.eurpolymj.2012.12.009

[50] Nishinari K. Some thoughts on the definition of a gel. In toyoichi tanaka memorial symposium on gels. Progr Colloid Polym Sci. (2009) 136:87–94. 10.1007/2882_2009_12

[51] Mezzenga R Fischer P. The self-assembly, aggregation and phase transitions of food protein systems in one, two and three dimensions. Rep Prog Phys. (2013) 76:046601. 10.1088/0034-4885/76/4/046601 23455715

[52] Clark AH Kavanagh GM Ross-Murphy SB. Globular protein gelation - theory and experiment. Food Hydrocoll. (2001) 15:383–400. 10.1016/S0268-005X(01)00042-X

[53] Mohammadian M Madadlou A. Technological functionality and biological properties of food protein nanofibrils formed by heating at acidic condition. Trends Food Sci Technol. (2018) 75:115–28. 10.1016/j.tifs.2018.03.013

[54] Cao YP Mezzenga R. Design principles of food gels. Nature Food. (2020) 1:106–18. 10.1038/s43016-019-0009-x 37127997

[55] Foegeding EA. Food biophysics of protein gels: a challenge of nano and macroscopic proportions. Food Biophys. (2006) 1:41–50. 10.1007/s11483-005-9003-y

[56] Cao YP Mezzenga R. Food protein amyloid fibrils: origin, structure, formation, characterization, applications and health implications. Adv Colloid Interfac. (2019) 269:334–56. 10.1016/j.cis.2019.05.002 31128463

[57] Bolisetty S Harnau L Jung JM Mezzenga R. Gelation, phase behavior, and dynamics of beta-lactoglobulin amyloid fibrils at varying concentrations and ionic strengths. Biomacromolecules. (2012) 13:3241–52. 10.1021/bm301005w 22924940

[58] Schmitt C Bovay C Vuilliomenet AM Rouvet M Bovetto L Barbar R. Multiscale characterization of individualized beta-lactoglobulin microgels formed upon heat treatment under narrow pH range conditions. Langmuir. (2009) 25:7899–909. 10.1021/la900501n 19594178

[59] Nicolai T Britten M Schmitt C. Beta-Lactoglobulin and WPI aggregates: formation, structure and applications. Food Hydrocoll. (2011) 25:1945–62. 10.1016/j.foodhyd.2011.02.006

[60] Zhang H Zhang F Wu J. Physically crosslinked hydrogels from polysaccharides prepared by freeze-thaw technique. React Funct Polym. (2013) 73:923–8. 10.1016/j.reactfunctpolym.2012.12.01424299774

[61] Figueroa-Pizano MD Velaz I Penas FJ Zavala-Rivera P Rosas-Durazo AJ Maldonado-Arce AD. Effect of freeze-thawing conditions for preparation of chitosan-poly (vinyl alcohol) hydrogels and drug release studies. Carbohydr Polym. (2018) 195:476–85. 10.1016/j.carbpol.2018.05.004 29805002

[62] Tanaka R Hatakeyama T Hatakeyama H. Formation of locust bean gum hydrogel by freezing-thawing. Polym Int. (1998) 45:118–26. 10.1002/(SICI)1097-0126(199801)45:1<118::AID-PI908>3.0.CO;2-T

[63] Zhao Y Shen W Chen ZG Wu T. Freeze-thaw induced gelation of alginates. Carbohydr Polym. (2016) 148:45–51. 10.1016/j.carbpol.2016.04.047 27185114

[64] Araujo D Rodrigues T Alves VD Freitas F. Chitin-glucan complex hydrogels: optimization of gel formation and demonstration of drug loading and release ability. Polymers (Basel). (2022) 14:785. 10.3390/polym14040785 35215701 PMC8877193

[65] McCann TH Guyon L Fischer P Day L. Rheological properties and microstructure of soy-whey protein. Food Hydrocoll. (2018) 82:434–41. 10.1016/j.foodhyd.2018.04.02335139479

[66] Zhu JZ Zhong L Song YZ Qian ZM Cao XY Huang Q. Preparation and characterization of pectin/chitosan beads containing porous starch embedded with doxorubicin hydrochloride: a novel and simple colon targeted drug delivery system. Food Hydrocoll. (2019) 95:562–70. 10.1016/j.foodhyd.2018.04.042

[67] Tang MX Zhu YD Li D Adhikari B Wang LJ. Rheological, thermal and microstructural properties of casein/κ-carrageenan mixed systems. LWT. (2019) 113:108296. 10.1016/j.lwt.2019.108296

[68] Hu X. Wang YM Zhang LL Xu M. Construction of self-assembled polyelectrolyte complex hydrogel based on oppositely charged polysaccharides for sustained delivery of green tea polyphenols. Food Chem. (2020) 306:125632. 10.1016/j.foodchem.2019.125632 31606634

[69] Le XT Rioux LE Turgeon SL. Formation and functional properties of protein-polysaccharide electrostatic hydrogels in comparison to protein or polysaccharide hydrogels. Adv Colloid Interface Sci. (2017) 239:127–35. 10.1016/j.cis.2016.04.006 27318757

[70] Li YQ Shi TF An LJ Huang QR. Monte carlo simulation on complex formation of proteins and polysaccharides. J Phys Chem B. (2012) 116:3045–53. 10.1021/jp206527p 22280485

[71] Laftah WA Hashim S Ibrahim AN. Polymer hydrogels: a review. Polym-Plast Technol Eng. (2011) 50:1475–86. 10.1080/03602559.2011.593082

[72] Bhattarai N Gunn J Zhang MQ. Chitosan-based hydrogels for controlled, localized drug delivery. Adv Drug Deliv Rev. (2010) 62:83–99. 10.1016/j.addr.2009.07.019 19799949

[73] Buenger D Topuz F Groll J. Hydrogels in sensing applications. Prog Polym Sci. (2012) 37:1678–719. 10.1016/j.progpolymsci.2012.09.001

[74] Botaro VR Santos CG Oliveira VA. Synthesis of hydrogels of cellulose acetate (AC) cross-linked with 3,3 ',4,4 ' benzophenonetetracarboxylic dianhydride (BTDA): characterization and adsorption physicochemical study. Polimeros. (2009) 19:278–84. 10.1590/S0104-14282009000400006

[75] Paulino AT Pereira AGB Fajardo AR Erickson K Kipper MJ Muniz EC. Natural polymer-based magnetic hydrogels: Potential vectors for remote-controlled drug release. Carbohydr Polym. (2012) 90:1216–25. 10.1016/j.carbpol.2012.06.051 22939334

[76] Chen Q Zhu L Zhao C Wang QM Zheng J. A robust, one-pot synthesis of highly mechanical and recoverable double network hydrogels using thermoreversible sol-gel polysaccharide. Adv Mater. (2013) 25:4171–6. 10.1002/adma.201300817 23765594

[77] Paulino AT Guilherme MR de Almeida EAMS Pereira AGB Muniz EC Tambourgi EB. One-pot synthesis of a chitosan-based hydrogel as a potential device for magnetic biomaterial. J Magn Magn Mater. (2009) 321:2636–42. 10.1016/j.jmmm.2009.03.078

[78] Bashir S Teo YY Ramesh S Ramesh K Mushtaq MW. Rheological behavior of biodegradable N-succinyl chitosan-g-poly (acrylic acid) hydrogels and their applications as drug carrier and in vitro theophylline release. Int J Biol Macromol. (2018) 117:454–66. 10.1016/j.ijbiomac.2018.05.182 29807081

[79] Lee J Lee BS Baik S Kim D Park NJ Lee JW. Ultra-intimate hydrogel hybrid skin patch with asymmetric elastomeric spatula-like cylinders. Chem Eng J. (2022) 444:136581.

[80] Li ZM Li G Xu JJ Li CW Han SL Zhang CF. Hydrogel transformed from nanoparticles for prevention of tissue injury and treatment of inflammatory diseases. Adv Mater. (2022) 34:2109178. 10.1002/adma.202109178 35195940

[81] Zhou Z Xiao JW Guan SW Geng ZJ Zhao RF Gao BT. hydrogen-bonded antibacterial curdlan-tannic acid hydrogel with an antioxidant and hemostatic function for wound healing. Carbohydr Polym. (2022) 285:119235. 10.1016/j.carbpol.2022.119235 35287859

[82] Wang M Wang T Luo YF He K Pan L Li Z. Fusing stretchable sensing technology with machine learning for human–machine interfaces. Adv Funct Mater. (2021) 31:2008807. 10.1002/adfm.202008807

[83] Zhang C Zhou YS Han HJ Zheng HX Xu WH Wang ZK. Dopamine-triggered hydrogels with high transparency, self-adhesion, and thermoresponse as skinlike sensors. ACS Nano. (2021) 15:1785–94. 10.1021/acsnano.0c09577 33404217

[84] Lee KY Mooney DJ. Hydrogels for tissue engineering. Chem Rev. (2001)101:1869–79. 10.1021/cr000108x 11710233

[85] Hoffman AS. Hydrogels for biomedical applications. Adv Drug Deliv Rev. (2002) 54:3–12. 10.1016/s0169-409x(01)00239-3 11755703

[86] Narayanaswamy R Torchilin VP. Hydrogels and their applications in targeted drug delivery. Molecules. (2019) 24:603. 10.3390/molecules24030603 30744011 PMC6384686

[87] Mao L Lu Y Cui MN Miao S Gao YX. Design of gel structures in water and oil phases for improved delivery of bioactive food ingredients. Crit Rev Food Sci Nutr. (2020) 60:1651–66. 10.1080/10408398.2019.1587737 30892058

[88] Cooper RC Yang H. Hydrogel-based ocular drug delivery systems: emerging fabrication strategies, applications, and bench-to-bedside manufacturing considerations. J Control Release. (2019) 306:29–39. 10.1016/j.jconrel.2019.05.034 31128143 PMC6629478

[89] Chen LY Remondetto GE Subirade M. Food protein-based materials as nutraceutical delivery systems. Trends Food Sci Technol. (2006) 17:272–83. 10.1016/j.tifs.2005.12.01131398015

[90] Zhang Z Zhang RJ Chen L Tong QY McClements DJ. Designing hydrogel particles for controlled or targeted release of lipophilic bioactive agents in the gastrointestinal tract. Eur Polym J. (2015) 72:698–716. 10.1016/j.eurpolymj.2015.01.013

[91] Pasqui D De Cagna M Barbucci R. Polysaccharide-based hydrogels: the key role of water in affecting mechanical properties. Polymers. (2012) 4:1517–34. 10.3390/polym4031517

[92] Wang K Nune KC Misra RDK. The functional response of alginate-gelatin-nanocrystalline cellulose injectable hydrogels toward delivery of cells and bioactive molecules. Acta Biomater. (2016) 36:143–51. 10.1016/j.actbio.2016.03.016 26971665

[93] Babaei J Khodaiyan F Mohammadian M. Effects of enriching with gellan gum on the structural, functional, and degradation properties of egg white heat-induced hydrogels. Int J Biol Macromol. (2019) 128:94–100. 10.1016/j.ijbiomac.2019.01.116 30682479

[94] Xu X Xia XF Zhang KY Rai A Li Z Zhao PC. Bioadhesive hydrogels demonstrating pH-independent and ultrafast gelation promote gastric ulcer healing in pigs. Sci Transl Med. (2020) 12:eaba8014. 10.1126/scitranslmed.aba8014 32848095

[95] Ozel B Aydin O Grunin L Oztop MH. Physico-chemical changes of composite whey protein hydrogels in simulated gastric fluid conditions. J Agric Food Chem. (2018) 66:9542–55. 10.1021/acs.jafc.8b02829 30111102

[96] Xue WL Yang R Liu S Pu YJ Wang PH Zhang WJ. Ascidian-inspired aciduric hydrogels with high stretchability and adhesiveness promote gastric hemostasis and wound healing. Biomater Sci. (2022) 10:2417–27. 10.1039/D2BM00183G 35393995

[97] Liu JY Pang Y Zhang SY Cleveland C Yin XL Booth L. Triggerable tough hydrogels for gastric resident dosage forms. Nat Commun. (2017) 8:124. 10.1038/s41467-017-00144-z 28743858 PMC5527117

[98] He JH Zhang ZX Yang YT Ren FG Li JP Zhu SJ. Injectable self-healing adhesive pH-responsive hydrogels accelerate gastric hemostasis and wound healing. Nano-Micro Lett. (2021) 13:80. 10.1007/s40820-020-00585-0 34138263 PMC8187506

[99] Don TM. King CF, Chiu WY. Synthesis and properties of chitosan-modified poly(vinyl acetate). J Appl Polym Sci. (2002) 86:3057–63. 10.1002/app.11329

[100] Das S Subuddhi U. Controlled and targeted delivery of diclofenac sodium to the intestine from pH-Responsive chitosan/poly(vinyl alcohol) interpenetrating polymeric network hydrogels. Polymer Science Series A. (2016) 58:154–66. 10.1134/S0965545X16020048

[101] Knipe JM Chen F Peppas NA. Enzymatic biodegradation of hydrogels for protein delivery targeted to the small intestine. Biomacromolecules. (2015) 16:962–72. 25674922 10.1021/bm501871a

[102] Liu L Zhang Y Yu SJ Yang ZM He CL Chen XS. Dual stimuli-responsive nanoparticle-incorporated hydrogels as an oral insulin carrier for intestine-targeted delivery and enhanced paracellular permeation. ACS Biomater Sci Eng. (2018) 4:2889–902. 10.1021/acsbiomaterials.8b00646 33435012

[103] Yamagata T Morishita M Kavimandan NJ Nakamura K Fukuoka Y Takayama K. Characterization of insulin protection properties of complexation hydrogels in gastric and intestinal enzyme fluids. J Control Release. (2006) 112:343–9. 10.1016/j.jconrel.2006.03.005 16631271

[104] Epstein J Sanderson IR MacDonald TT. Curcumin as a therapeutic agent: the evidence from in vitro. Br J Nutr. (2010) 103:1545–57. 10.1017/S0007114509993667 20100380

[105] Hu B Shen Y Adamcik J Fischer P Schneider M Loessner M J. Polyphenol-binding amyloid fibrils self-assemble into reversible hydrogels with antibacterial activity. ACS Nano. (2018) 12:3385–96. 10.1021/acsnano.7b08969 29553709

[106] Hu B Yu SJ Shi C Gu J Shao Y Che Q. Amyloid-polyphenol hybrid nanofilaments mitigate colitis and regulate gut microbial dysbiosis. ACS Nano. (2020) 14:2760–76. 10.1021/acsnano.9b09125 31961657

[107] Hu B Li M He XQ Wang HL Huang JA Liu ZH. Flavonoid-amyloid fibril hybrid hydrogels for obesity control via construction of gut microbiota. Biomat Sci. (2022) 10:3597–611. 10.1039/D2BM00366J 35642606

[108] Taheri A Jafari SM. Science, gum-based nanocarriers for the protection and delivery of food bioactive compounds - sciencedirect. Adv Colloid Interface Sci. (2019) 269:277–95. 10.1016/j.cis.2019.04.009 31132673

[109] Wang ZG Zhang RX Zhang C Dai CX Ju XR He R. Fabrication of stable and self-assembling rapeseed protein nanogel for hydrophobic curcumin delivery. J Agric Food Chem. (2019) 67:887–94. 10.1021/acs.jafc.8b05572 30608682

[110] Alavi F Emam-Djomeh Z Yarmand MS Salami M Momen S Moosavi-Movahedi AA. Cold gelation of curcumin loaded whey protein aggregates mixed with k-carrageenan: impact of gel microstructure on the gastrointestinal fate of curcumin. Food Hydrocoll. (2018) 85:267–80. 10.1016/j.foodhyd.2018.07.012

[111] Hu M Du XQ Liu GN. Huang, YY, Qi BK, Li Y. Sodium alginate/soybean protein-epigallocatechin-3-gallate conjugate hydrogel beads: evaluation of structural, physical, and functional properties. Food Funct. (2021) 12:12347–61. 10.1039/D1FO03099J 34842261

[112] Liu W Kong YY Ye AQ Shen P Dong L Xu XK. Preparation, formation mechanism and in vitro dynamic digestion behavior of quercetin-loaded liposomes in hydrogels. Food Hydrocoll. (2020) 104:105743. 10.1016/j.foodhyd.2020.105743

[113] Hu X Wang YM Zhang LL Xu M. Formation of self-assembled polyelectrolyte complex hydrogel derived from salecan and chitosan for sustained release of Vitamin C. Carbohydr Polym. (2020) 234:115920. 10.1016/j.carbpol.2020.115920 32070539

[114] O'Neill GJ Jacquier JC Mukhopadhya A Egan T O'Sullivan M Sweeney T. In vitro and in vivo evaluation of whey protein hydrogels for oral delivery of riboflavin. J Funct Food. (2015) 19:512–21. 10.1016/j.jff.2015.09.043

[115] Camacho DH Uy SJY Cabrera MJF Lobregas MOS Fajardo TJMC. Encapsulation of folic acid in copper-alginate hydrogels and it's slow in vitro release in physiological pH condition. Food Res Int. (2019) 119:15–22. 10.1016/j.foodres.2019.01.053 30884643

[116] Pour PK Alemzadeh I Vaziri AS Beiroti A. Potential effects of alginate-pectin biocomposite on the release of folic acid and their physicochemical characteristics. J Food Sci Technol-Mysore. (2020) 57:3363–70. 10.1007/s13197-020-04369-7 32728283 PMC7374679

[117] Liu K Kong XL Li QM Zhang HL Zha XQ Luo JP. Stability and bioavailability of vitamin D3 encapsulated in composite gels of whey protein isolate and lotus root amylopectin. Carbohydr Polym. (2020) 227:115337. 10.1016/j.carbpol.2019.115337 31590880

[118] Somchue W Sermsri W Shiowatana J Siripinyanond A. Encapsulation of α-tocopherol in protein-based delivery particles. Food Res Int. (2009) 42:909–14. 10.1016/j.foodres.2009.04.021

[119] Dafe A Etemadi H Dilmaghani A Mandavinia GR. Investigation of pectin/starch hydrogel as a carrier for oral delivery of probiotic bacteria. Int J Biol Macromol. (2017) 97:536–43. 10.1016/j.ijbiomac.2017.01.060 28108413

[120] Huang X Ganzle M Zhang H Zhao M Fang YP Nishinari K. Microencapsulation of probiotic lactobacilli with shellac as moisture barrier and to allow controlled release. J Sci Food Agric. (2021) 101:726–34. 10.1002/jsfa.10685 32706117

[121] Su J Cai Y Zhi Z Guo Q Meeren PVD. Assembly of propylene glycol alginate/beta-lactoglobulin composite hydrogels induced by ethanol for co-delivery of probiotics and curcumin. Carbohydr Polym. (2021) 254:117446. 10.1016/j.carbpol.2020.117446 33357916

[122] Tripathi MK Giri SK. Probiotic functional foods: Survival of probiotics during processing and storage. J Funct Food. (2014) 9:225–41. 10.1016/j.jff.2014.04.03032036930

[123] Garcia-Brand AJ Quezada V Gonzalez-Melo C Bolanos-Barbosa AD Cruz JC Reyes LH. Novel developments on stimuli-responsive probiotic encapsulates: from smart hydrogels to nanostructured platforms. Fermentation. (2022) 8:117. 10.3390/fermentation8030117

[124] Kwiecien I Kwiecien M. Application of polysaccharide-based hydrogels as probiotic delivery systems. Gels. (2018) 4:47. 10.3390/gels4020047 30674823 PMC6209284

[125] Sun QS Wicker L. Hydrogel encapsulation of lactobacillus casei by block charge modified pectin and improved gastric and storage stability. Foods. (2021) 10:1337. 10.3390/foods10061337 34200620 PMC8227579

[126] Afzaal M Saeed F Hussain M Ismail Z Siddeeg A Al-Farga A. Influence of encapsulation on the survival of probiotics in food matrix under simulated stress conditions. Saudi J Biol Sci. (2022) 29:103394. 10.1016/j.sjbs.2022.103394 35942164 PMC9356273

[127] Wang M Zang YP Hong KJ Zhao XF Yu CR Liu DD. Preparation of pH-sensitive carboxymethyl cellulose/chitosan/alginate hydrogel beads with reticulated shell structure to deliver Bacillus subtilis natto. Int J Biol Macromol. (2021) 192:684–91. 10.1016/j.foodhyd.2021.107478 34648802

[128] Zhao M Huang X Zhang H Zhang YZ Ganzle M Yang N. Probiotic encapsulation in water-in-water emulsion via heteroprotein complex coacervation of type-A gelatin/sodium caseinate. Food Hydrocoll. (2020) 105:105790. 10.1016/j.foodhyd.2020.105790

[129] Gao HX Ma L Sun WX McClements DJ Cheng C Zeng HY. Impact of encapsulation of probiotics in oil-in-water high internal phase emulsions on their thermostability and gastrointestinal survival. Food Hydrocoll. (2022) 126:107478.

[130] Wang R Guo K Zhang WJ He YN Yang K Chen Q. Poly-γ-glutamic acid microgel-encapsulated probiotics with gastric acid resistance and smart inflammatory factor targeted delivery performance to ameliorate colitis. Adv Funct Mater. (2022) 26:2113034. 10.1002/adfm.202113034

[131] Lin YH Chang CH Wu YS Hsu YM Chiou SF Chen YJ. Development of pH-responsive chitosan/heparin nanoparticles for stomach-specific anti-Helicobacter pylori therapy. Biomaterials. (2009) 30:3332–42. 10.1016/j.biomaterials.2009.02.036 19299008

[132] Lin YH Tsai SC Lai CH Lee CH He ZS Tseng GC. Genipin-cross-linked fucose-chitosan/heparin nanoparticles for the eradication of Helicobacter pylori. Biomaterials. (2013) 34:4466–79. 10.1016/j.biomaterials.2013.02.028 23499480

[133] Liu H Cai ZW Wang F Hong LW Deng LF Zhong J. Colon-targeted adhesive hydrogel microsphere for regulation of gut immunity and flora. Adv Sci. (2021) 8:e2101619. 10.1002/advs.202101619 34292669 PMC8456273

[134] Praveschotinunt P Duraj-Thatte AM Gelfat I Bahl F Chou DB Joshi NS. Engineered E. coli Nissle 1917 for the delivery of matrix-tethered therapeutic domains to the gut. Nat Commun. (2019) 10:5580. 10.1038/s41467-019-13336-6 31811125 PMC6898321

[135] Duraj-Thatte AM Courchesne NMD Praveschotinunt P Rutledge J Lee Y Karp JM. Genetically programmable self-regenerating bacterial hydrogels. Adv Mater. (2019) 31:e1901826. 10.1002/adma.201901826 31402514 PMC6773506

[136] Wang X Cao ZP Zhang MM Meng L Ming ZZ Liu JY. Bioinspired oral delivery of gut microbiota by self-coating with biofilms. Sci Adv. (2020) 6:eabb1952. 10.1126/sciadv.abb1952 32637620 PMC7314526

[137] Mettu S Hathi Z Athukoralalage S Priya A Lam TN Ong KL. Perspective on constructing cellulose-hydrogel-based gut-like bioreactors for growth and delivery of multiple-strain probiotic bacteria. J Agri Food Chem. (2021) 69:4946–59. 10.1021/acs.jafc.1c00468 33890783 PMC8154558

